# Supplementary material for: Multifaceted analysis of cross-tissue transcriptomes reveals phenotype–endotype associations in atopic dermatitis
Source: Nat Commun. 2023 Oct 2;14:6133. doi: 10.1038/s41467-023-41857-8 (PMC10545679; doi:10.1038/s41467-023-41857-8)
Supplement: Supplementary file 1 — Supplementary Information [file 41467_2023_41857_MOESM1_ESM.pdf]

Multifaceted analysis of cross-tissue transcriptomes reveals phenotype–endotype associations in atopic dermatitis.

Sekita et al. 2023

Supplementary information

Supplementary Figures

Supplementary Tables

Supplementary Notes

Supplementary Figures

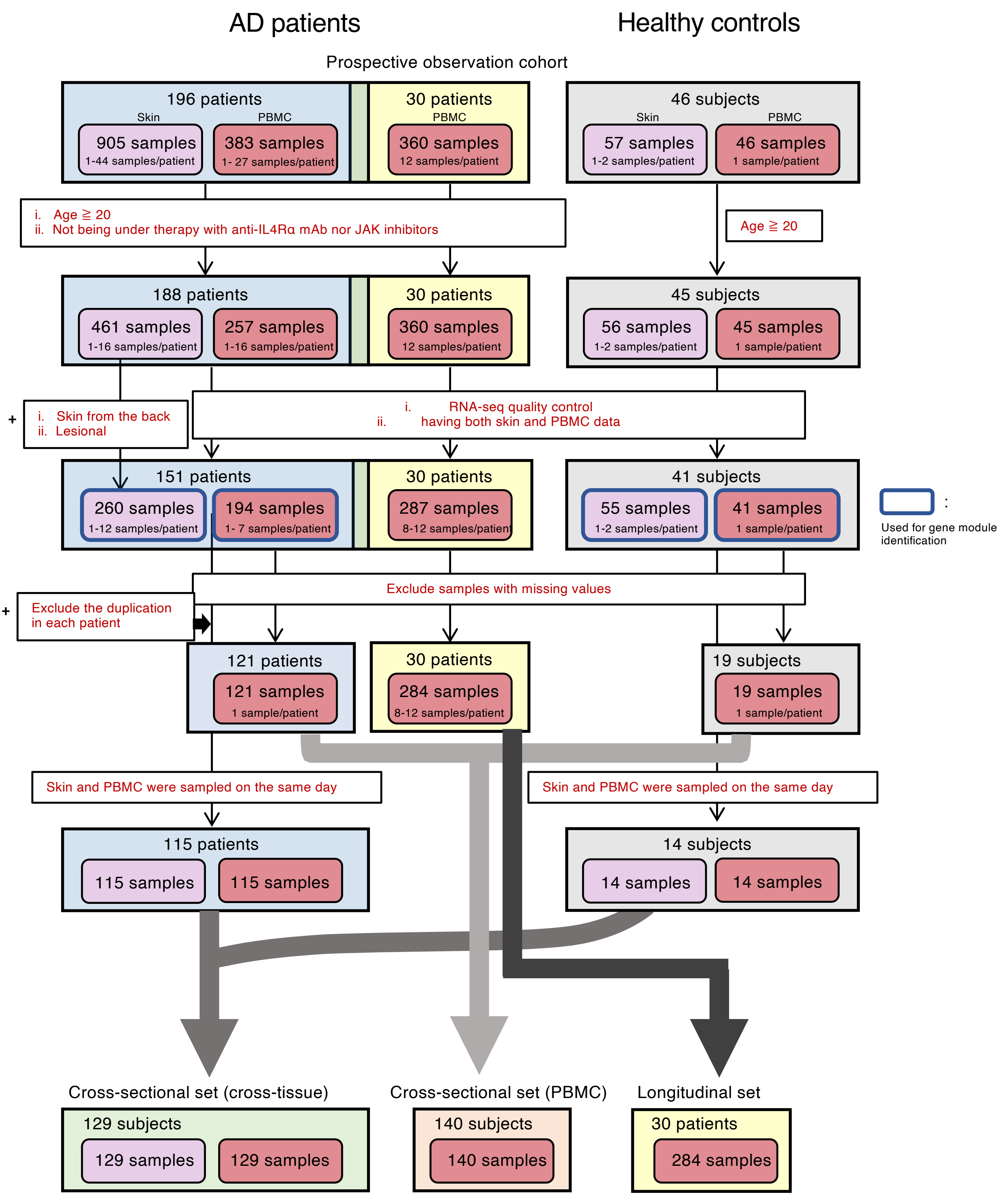

Supplementary Figure 1 A schematic presentation showing the process of filtering sample and patient for each analysis.

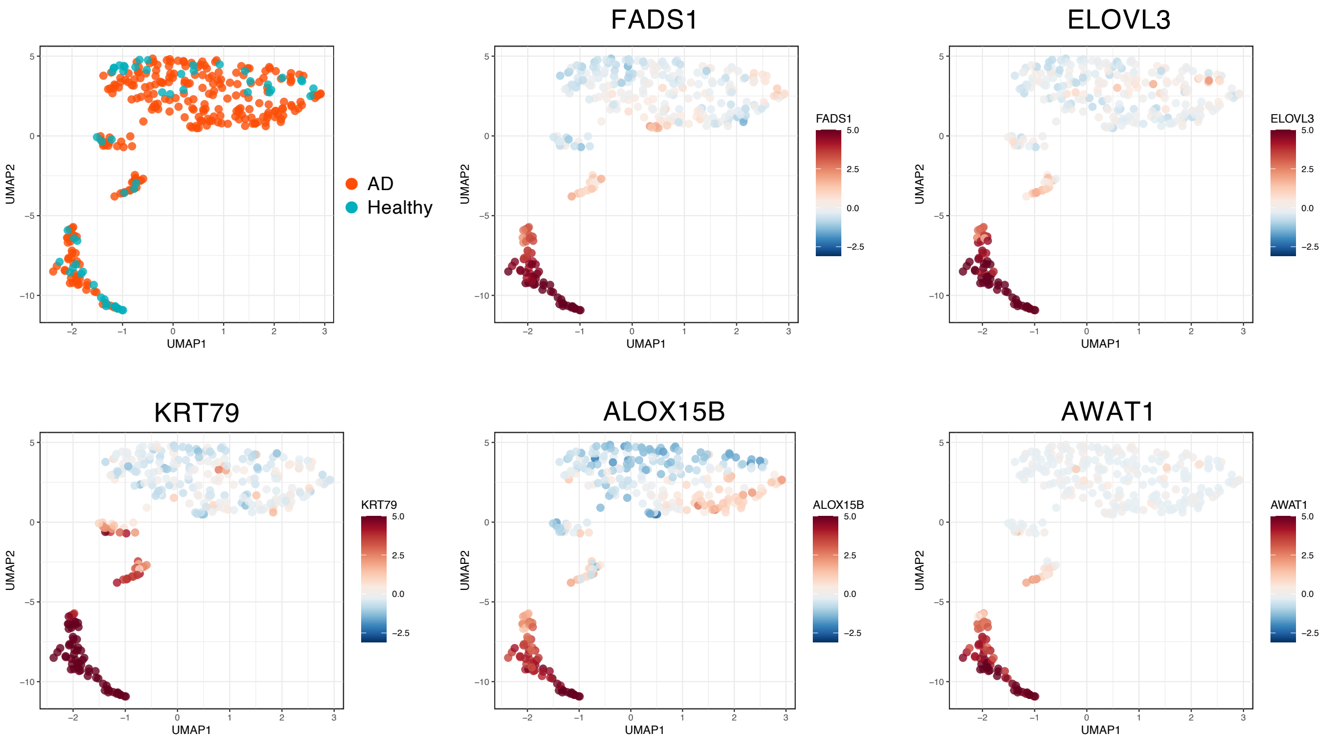

Supplementary Figure 2 Skin sample filtering by expression of hair follicle-related genes.
UMAP plot of expression of pilosebaceous unit-related genes in skin samples from both AD patients and healthy controls. Sixty-five skin samples (AD: 45, healthy controls: 20) forming a cluster with extremely strong signatures of the hair follicle gene set were considered to be occupied by hair follicles along with incidental sebaceous glands, and therefore excluded from this study.

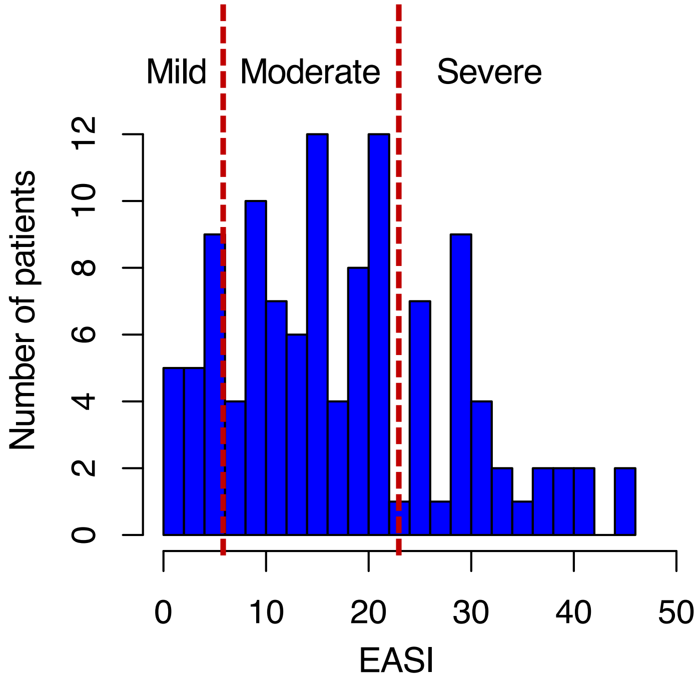

Supplementary Figure 3 Histogram of frequency of AD patients at different disease severity. The histogram shows the distribution of AD patients in cross-sectional analysis at different scores of EASI. Severity strata was defined using EASI in individual patients; mild: 0.1 – 5.9, moderate: 6.0 – 22.9, severe: 23-72. Source data are provided as a Source Data file.

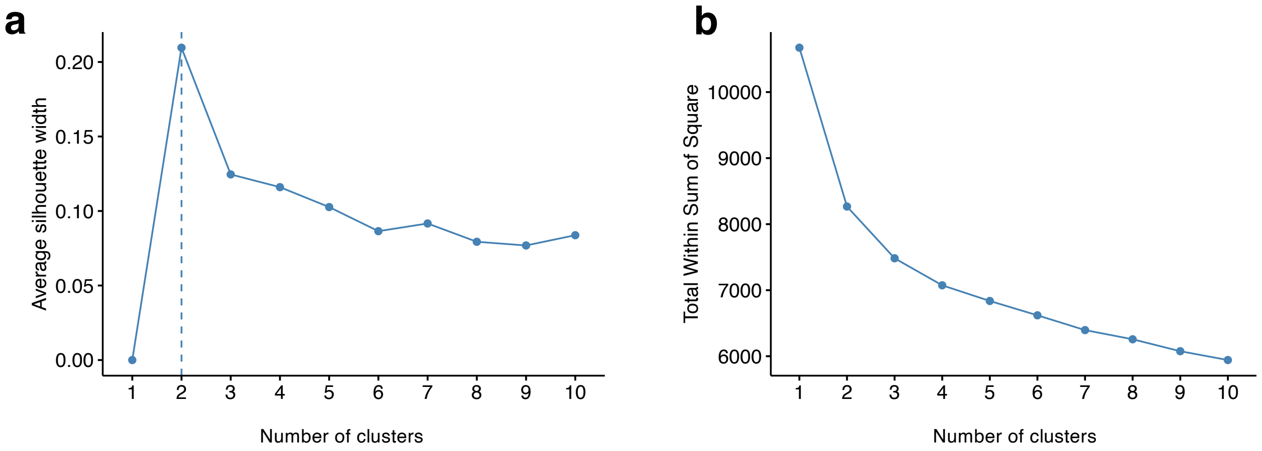

Supplementary Figure 4 Identification of the optimal number of clusters for individual EASI partial scores.

a,b. Silhouette width plot (**a**) and elbow plot (**b**) for identifying the optimal number of clusters for the correlation matrix of EASI partial score. The X-axes indicate the number of clusters ranging from 1 to 10 and the Y-axes indicate average silhouette width (**a**) or total within sum of square (**b**). Both of a highest average silhouette coefficient in the silhouette plot and a large drop in the elbow plot were observed at the number of clusters of 2.

a

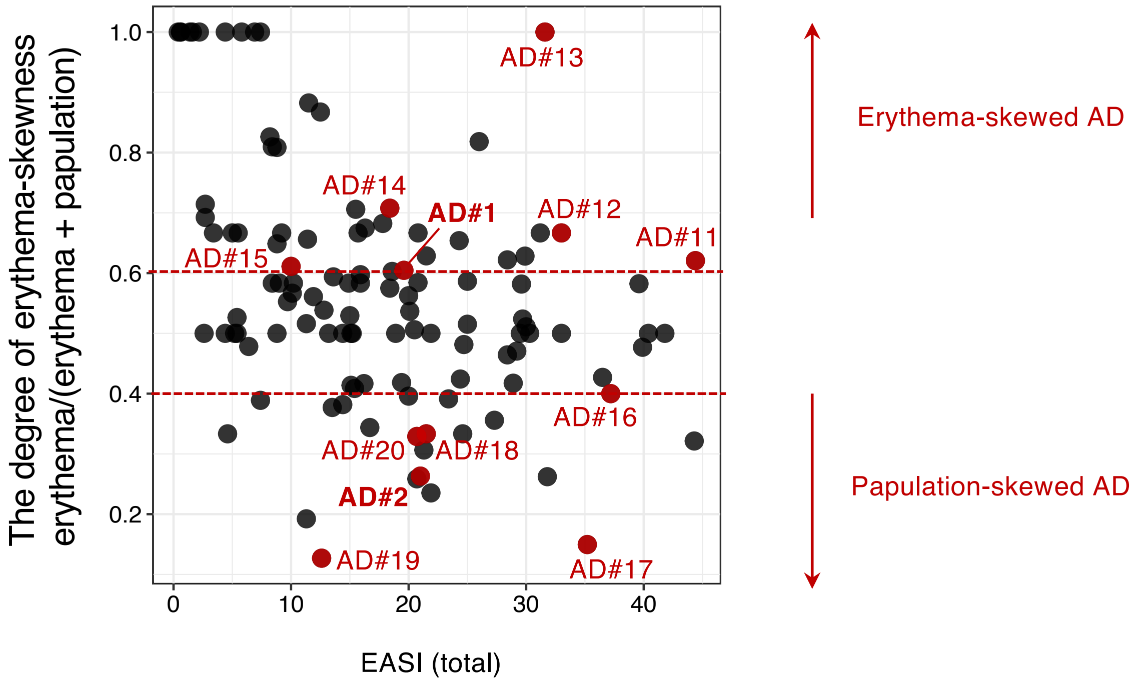

The degree of erythema-skewness
erythema/(erythema + papulation)
1.0
0.8
0.6
0.4
0.2
0
10
20
30
40
EASI (total)
AD#13
AD#14
AD#1
AD#12
AD#11
AD#15
AD#16
AD#20
AD#18
AD#2
AD#19
AD#17
Erythema-skewed AD
Papulation-skewed AD

b

(Erythema-skewed AD)

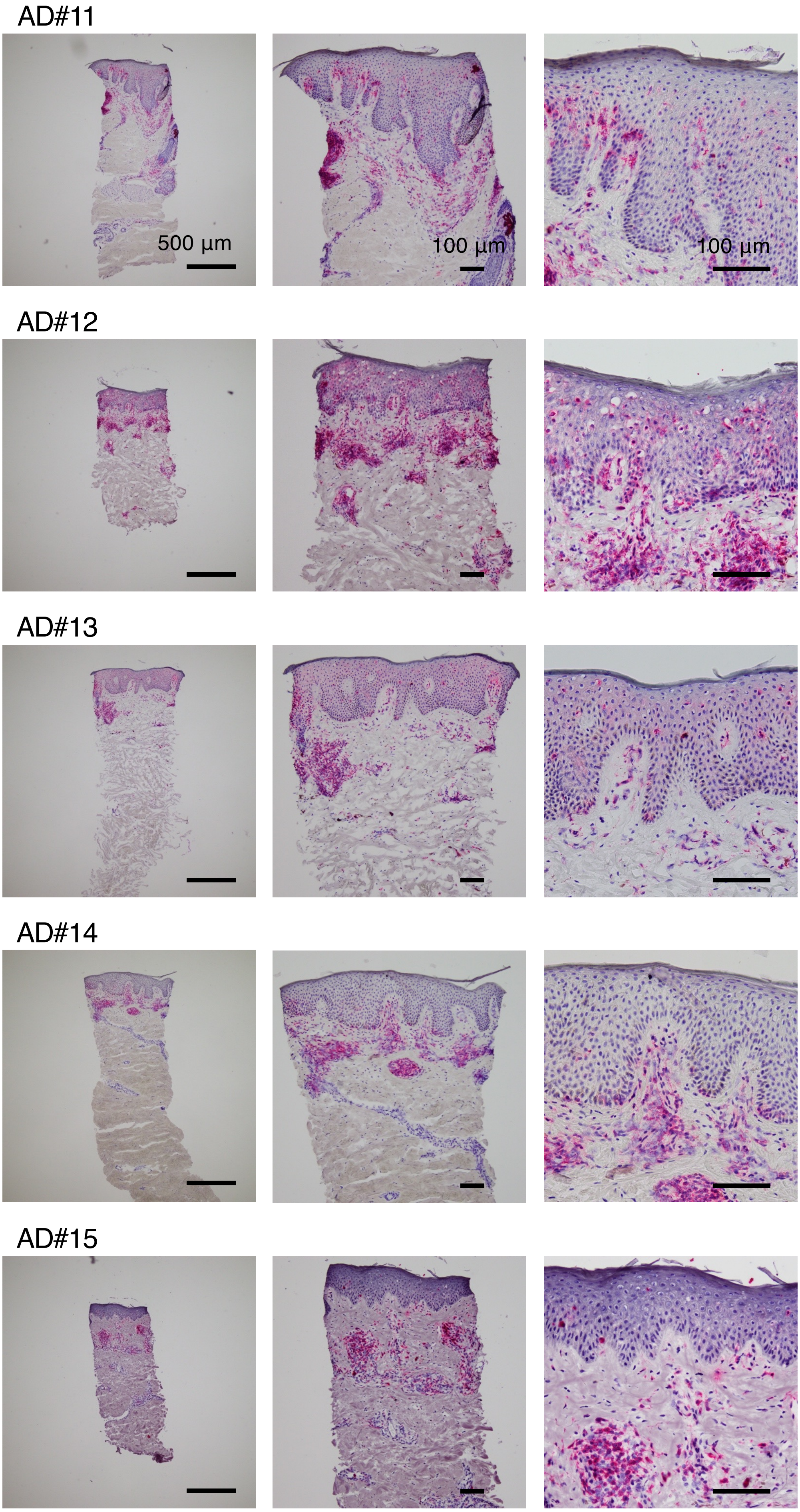

c

(Papulation-skewed AD)

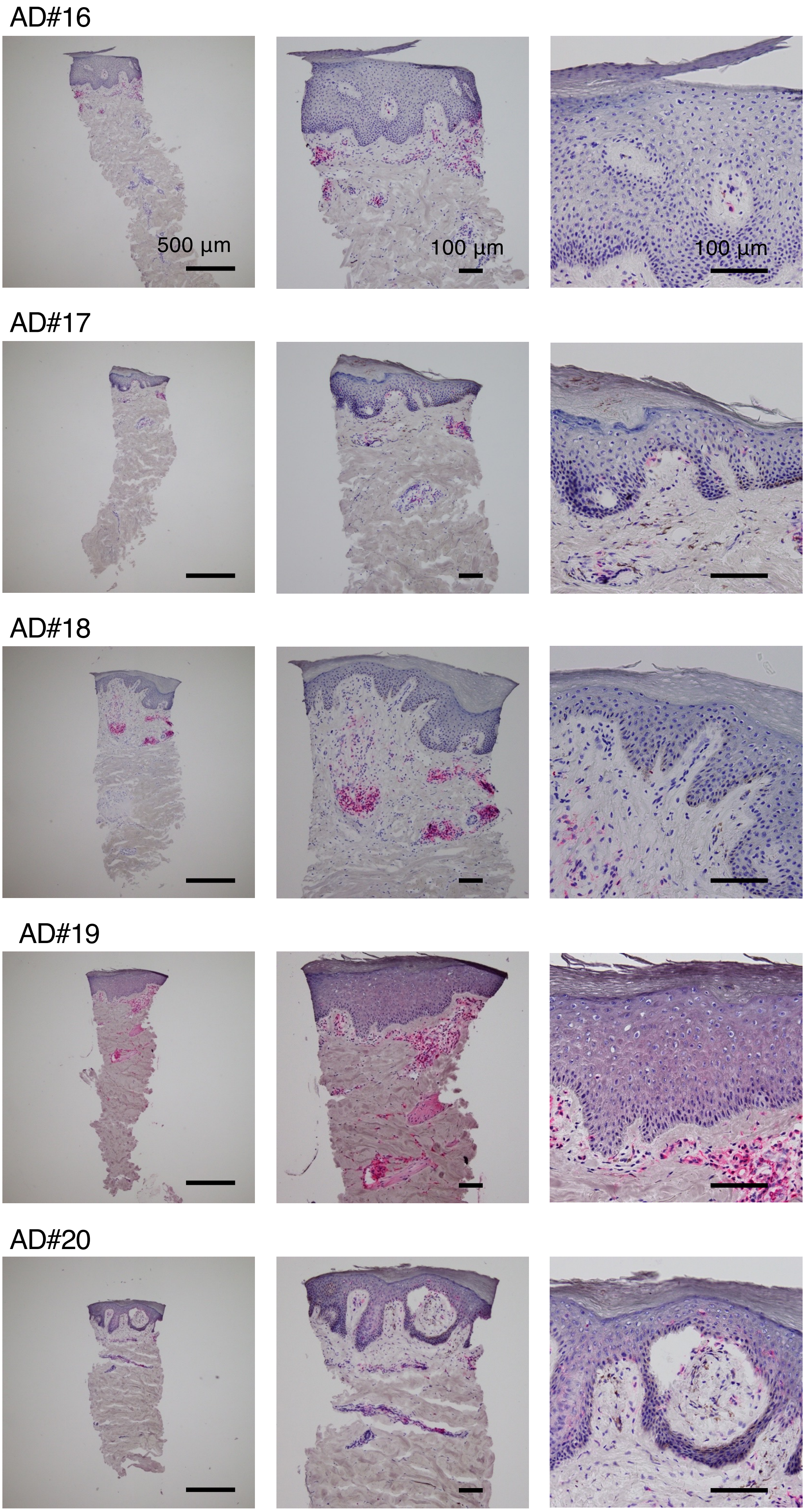

Supplementary Figure 5 Immunohistochemistry of skin tissue from the AD patients who have either erythema- or papulation-skewed skin manifestations.

a. We defined the degree of erythema-skewness as erythema/(erythema + papulation) using EASI partial points, and randomly picked 5 patients who have erythema-skewness $\geqq 0.6$ as erythema-skewed patients and 5 patients who have erythema-skewness $\leqq 0.4$ as papulation-skewed patients. **b,c.** Immunohistochemistry of skin tissue stained for CD4 (target protein was stained in red) in 5 erythema-skewed patients (**b**) and 5 papulation-skewed patients (**c**) picked. Bars: 500 µm (the right column), 100 µm (the middle and left columns). One slide per patient was assayed for one marker protein in histological analysis.

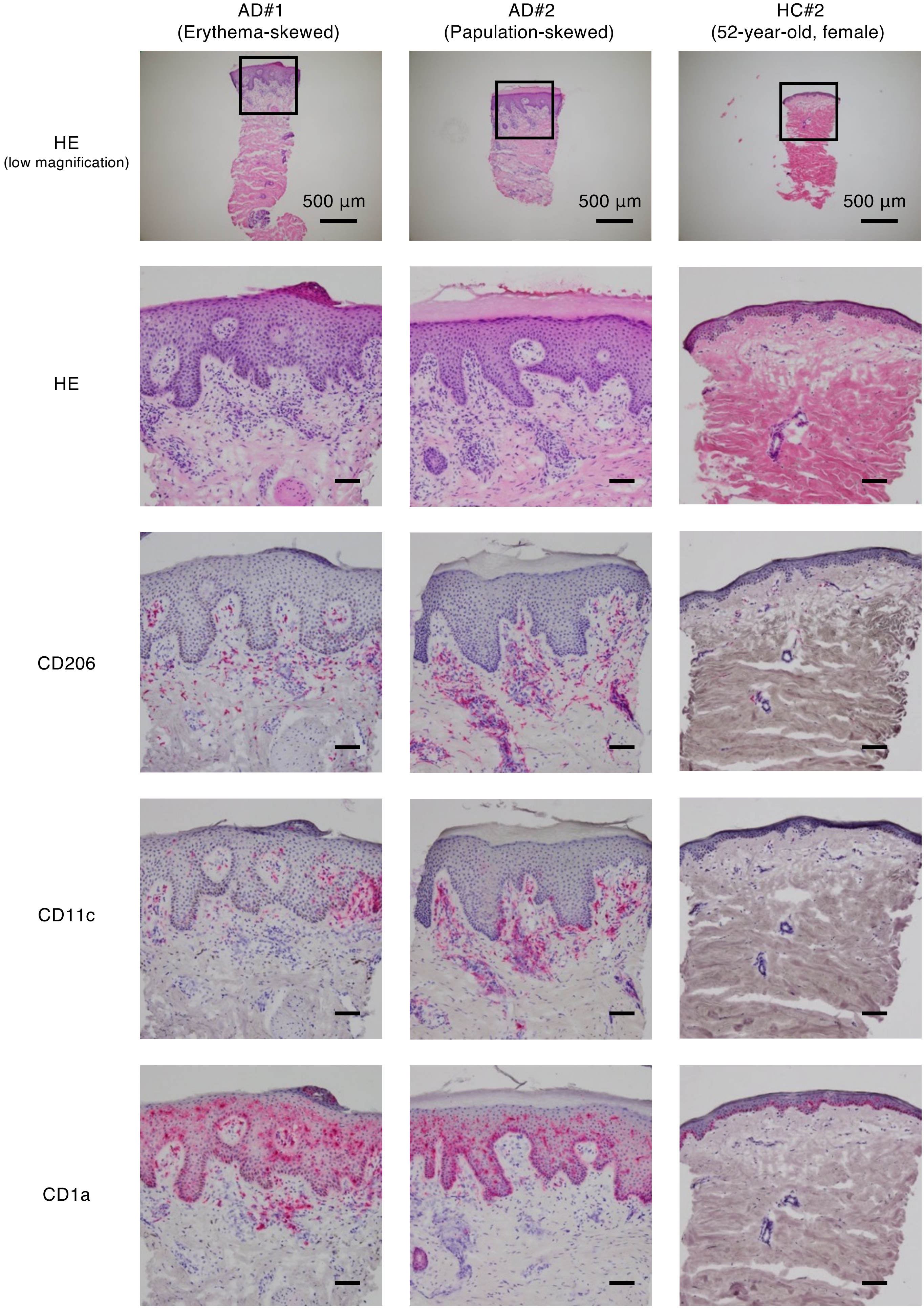
AD#1
(Erythema-skewed)
AD#2
(Papulation-skewed)
HC#2
(52-year-old, female)
HE
(low magnification)
500 µm
500 µm
500 µm
HE
CD206
CD11c
CD1a

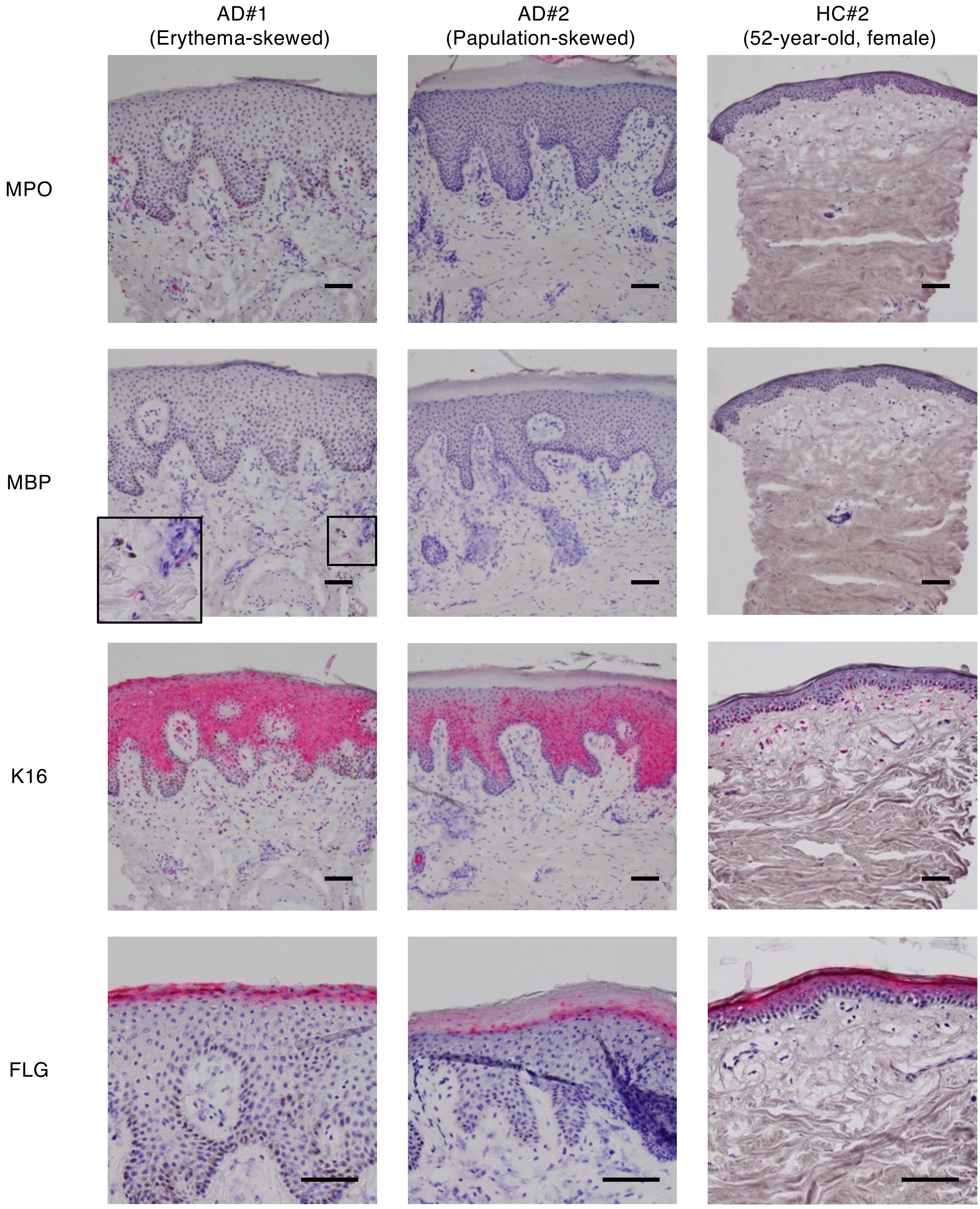

Supplementary Figure 6 Immunohistochemical analysis revealed shared and differential characteristics in the skin tissue of erythema and papulation-skewed AD patients. Immunohistochemistry of skin tissue in two representative AD patients who have a score composition that are highly skewed to either of erythema (upper) or induration/papulation (lower) as well as healthy control. Left: a 51-year-old male patient who has erythema-skewed EASI composition. Middle: a 50-year-old male patient who has papulation-skewed EASI composition (total = 21.0, erythema = 3.0, papulation = 8.4). Right: a 52-year-old female who have no eczema nor skin diseases. Target proteins were stained in red. Bars = 100 µm (except for low magnification HE images). MPO: myeloperoxidase, MBP: major basic protein, K16: keratin-16, FLG: filaggrin. One slide per patient was assayed for one marker protein in histological analysis.

a

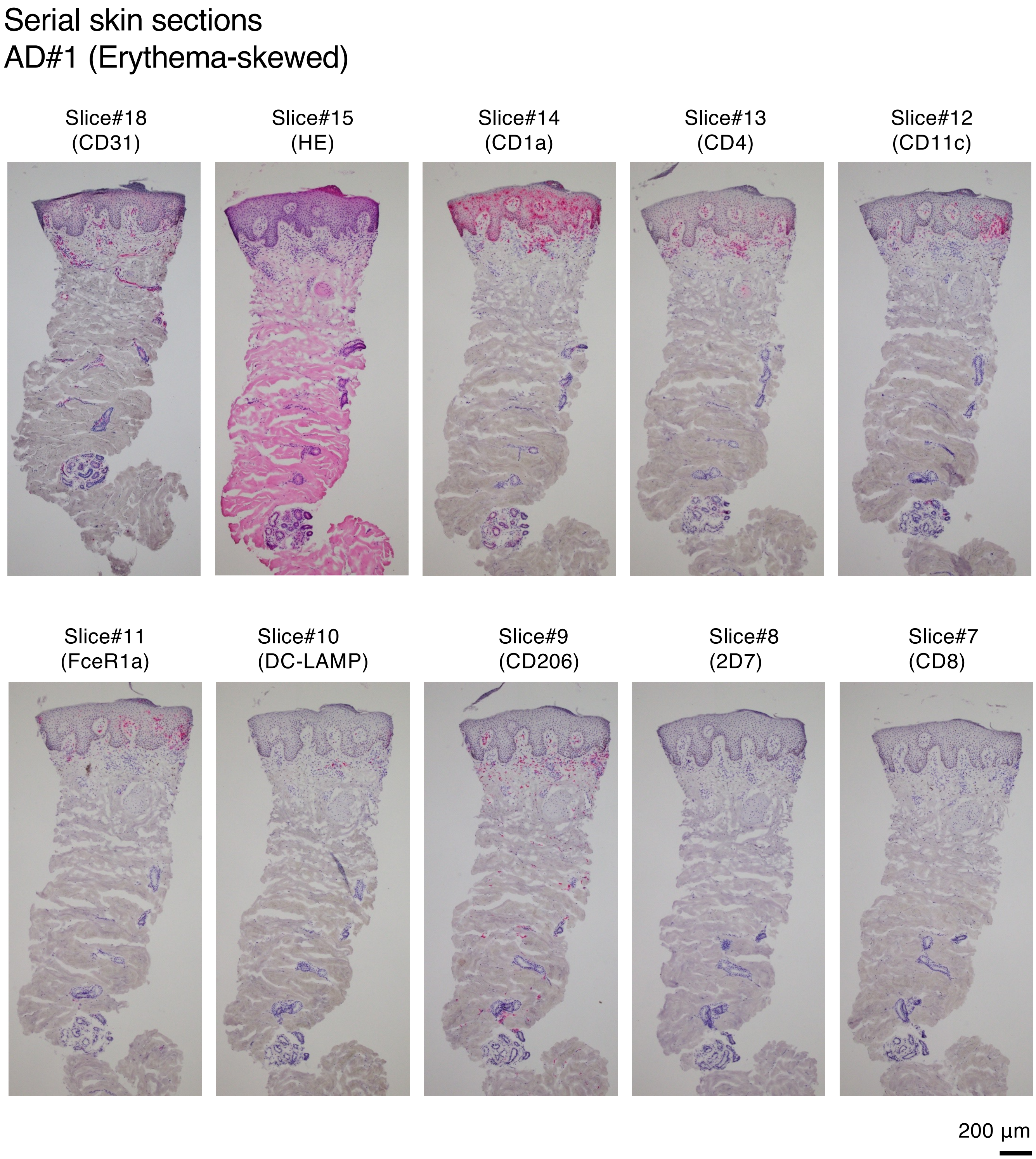
Serial skin sections
AD#1 (Erythema-skewed)
Slice#18
(CD31)
Slice#15
(HE)
Slice#14
(CD1a)
Slice#13
(CD4)
Slice#12
(CD11c)
Slice#11
(FceR1a)
Slice#10
(DC-LAMP)
Slice#9
(CD206)
Slice#8
(2D7)
Slice#7
(CD8)
200 µm

b

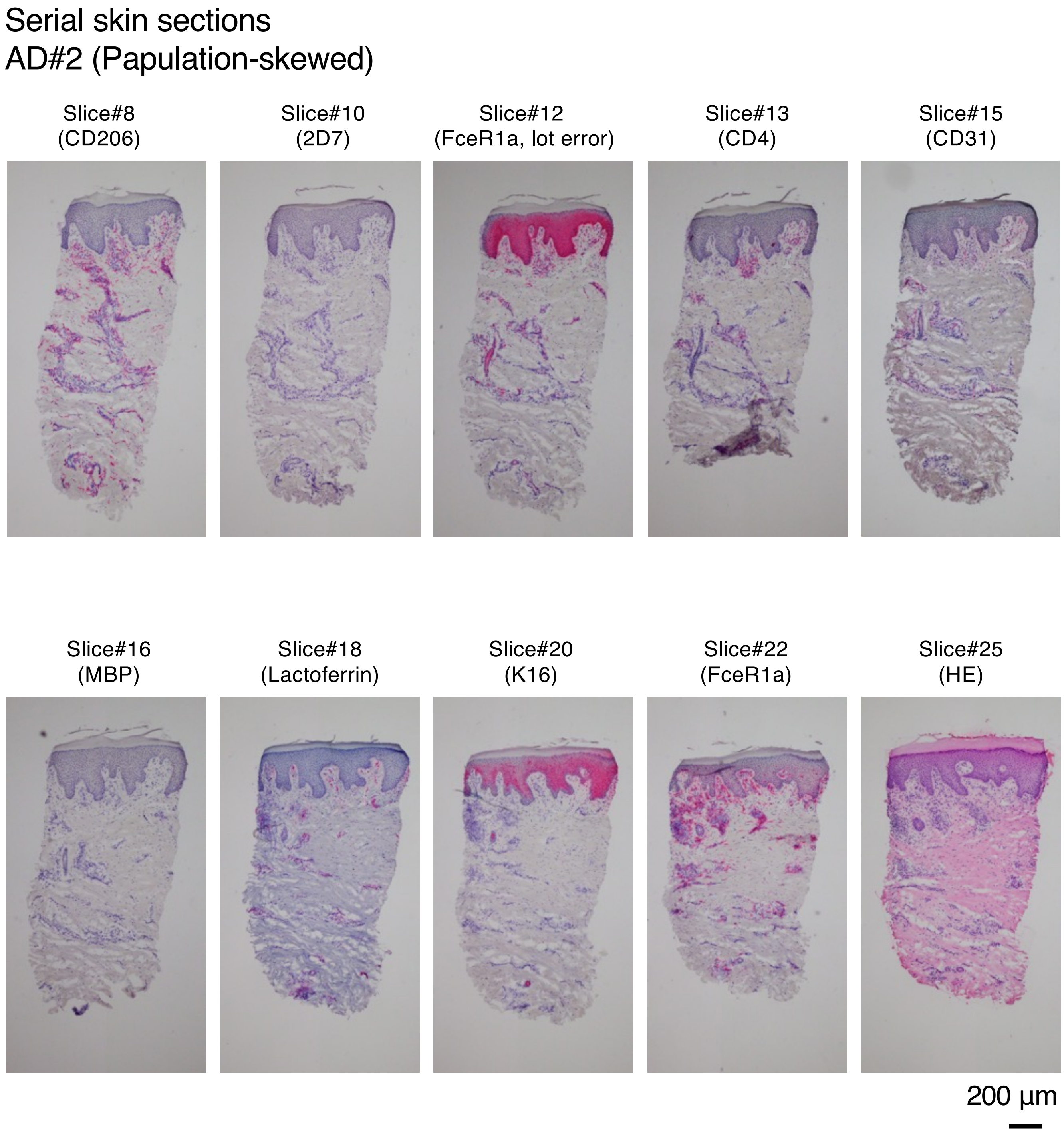

Supplementary Figure 7 Serial sections of skin specimen from the AD patients who have either erythema- or papulation-skewed skin manifestations.
Different kinds of immunohistochemistry were performed on serial sections from the representative erythema-skewed AD patient (**a**, AD#1) and papulation-skewed AD patient (**b**, AD#2). One slide per patient was assayed for one marker protein in histological analysis. MBP: major basic protein, K16: keratin-16.

Multiple comparison among AD strata (Kruskal-Wallis test):

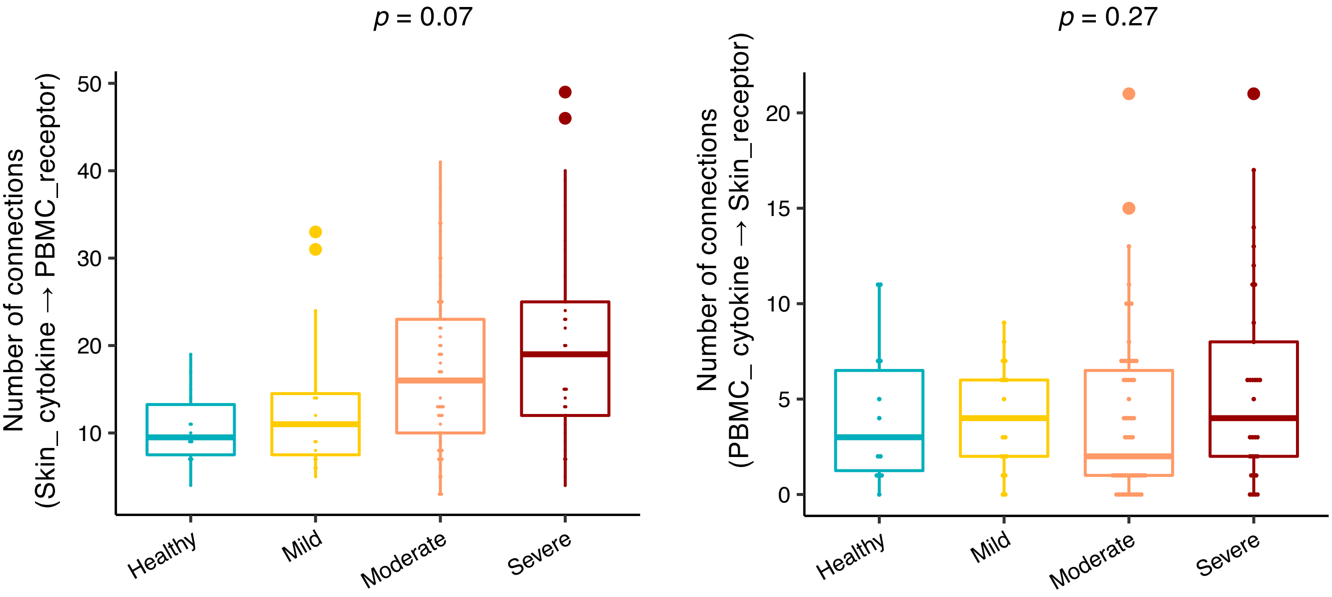

Supplementary Figure 8 Comparison of number of active connections between skin and PBMC among three AD severity strata.
The number of active connections were assessed based on cytokine – receptor coupling. Severity strata was defined using EASI in individual patients; mild: 0.1 – 5.9, moderate: 6.0 – 22.9, severe: 23-72. Multiple comparison tests were carried out on three AD severity strata with Kruskal-Wallis test. Boxplots show median and first and third quartiles, whiskers extending to the highest and lowest values no further than 1.5*interquartile range. N; Healthy = 14, Mild = 19, Mild = 63, Severe = 33 (biologically independent samples). Source data are provided as a Source Data file.

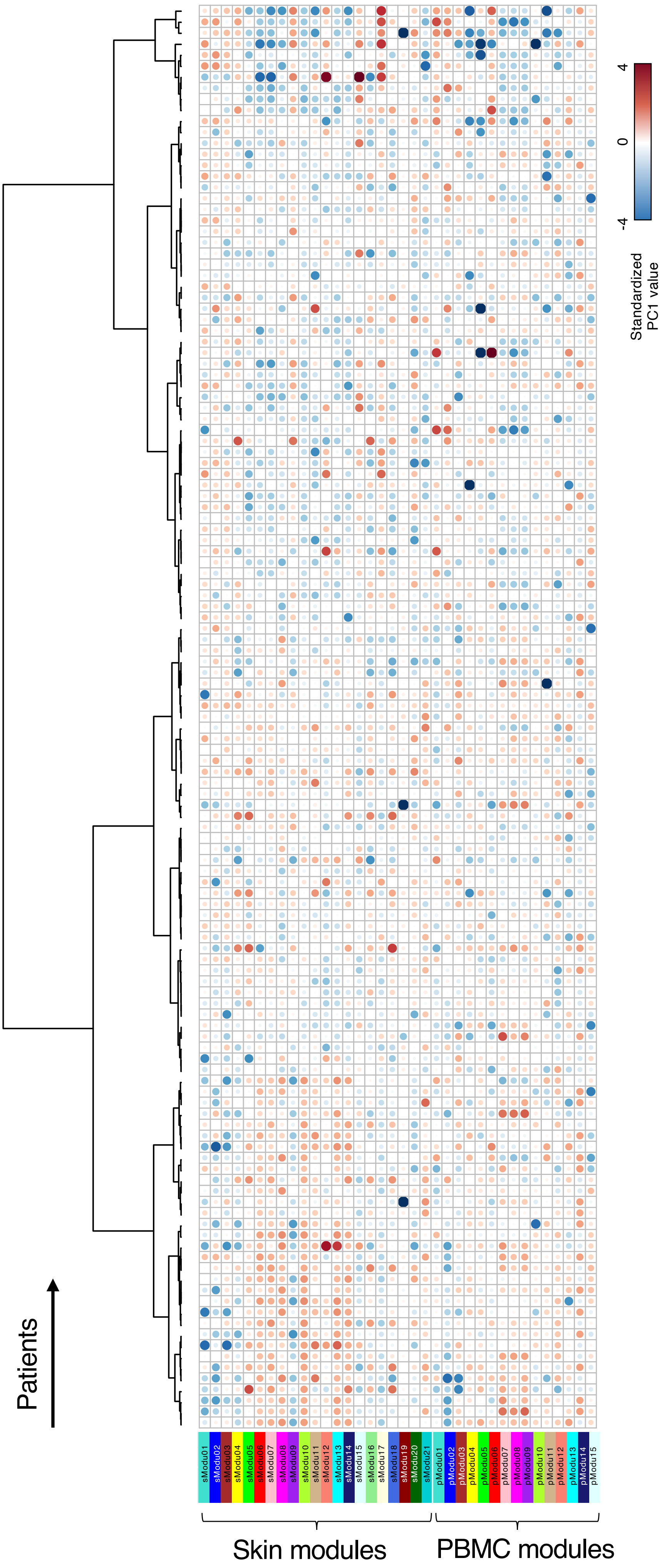

Patients
Standardized PC1 value
4
0
-4
sModu01
sModu02
sModu03
sModu04
sModu05
sModu06
sModu07
sModu08
sModu09
sModu10
sModu11
sModu12
sModu13
sModu14
sModu15
sModu16
sModu17
sModu18
sModu19
sModu20
sModu21
pModu01
pModu02
pModu03
pModu04
pModu05
pModu06
pModu07
pModu08
pModu09
pModu10
pModu11
pModu12
pModu13
pModu14
pModu15
Skin modules
PBMC modules

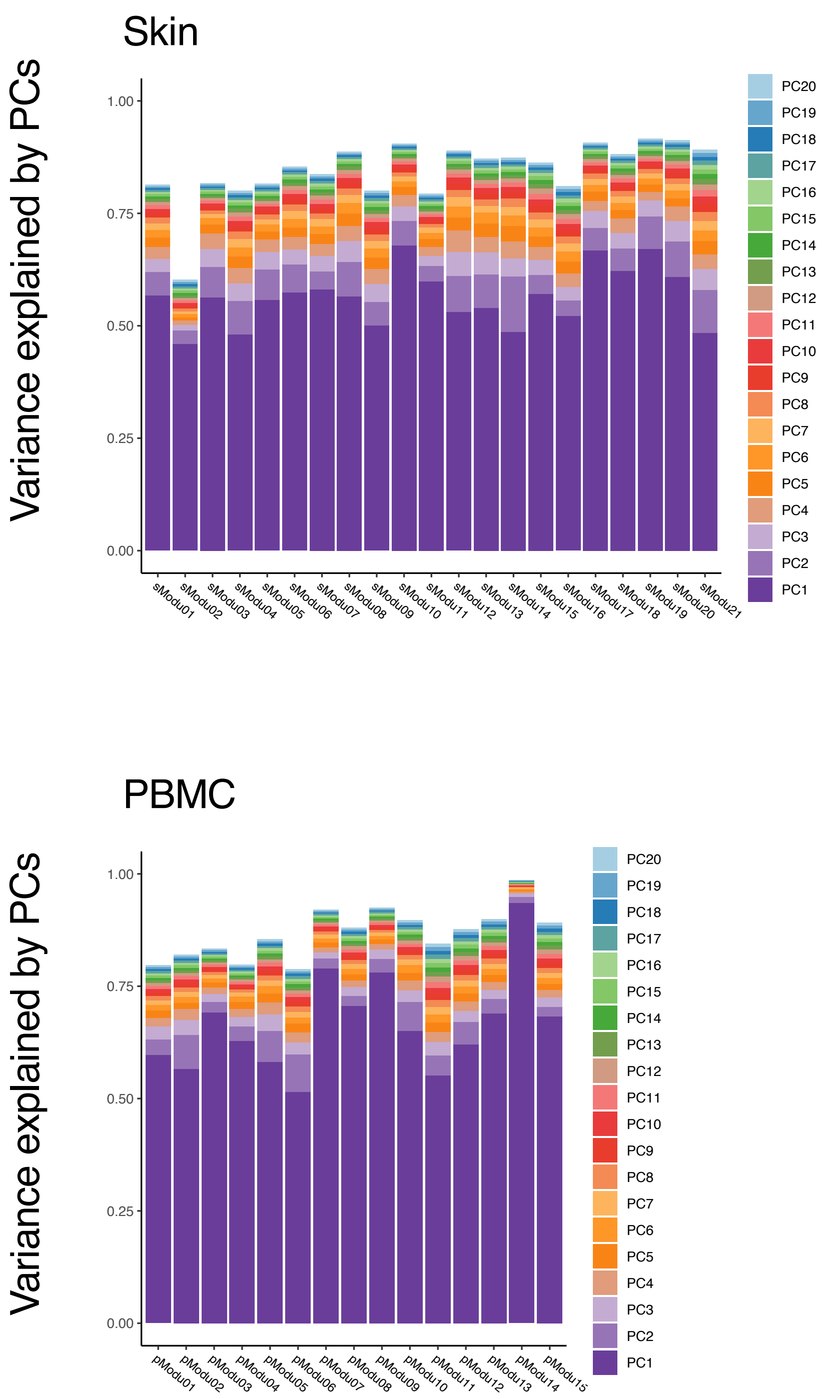

Supplementary Figure 9 Statistics of transcriptome modules associated with AD.
a. A plot shows the size of PC1 value per patient. PC1 value were obtained by applying PCA on gene expression data of patients for each gene module. **b.** Variance explained by PC1-PC20 in PCA on expression level of transcriptome modules in skin (upper) and PBMC (lower) across patients. Source data are provided as a Source Data file.

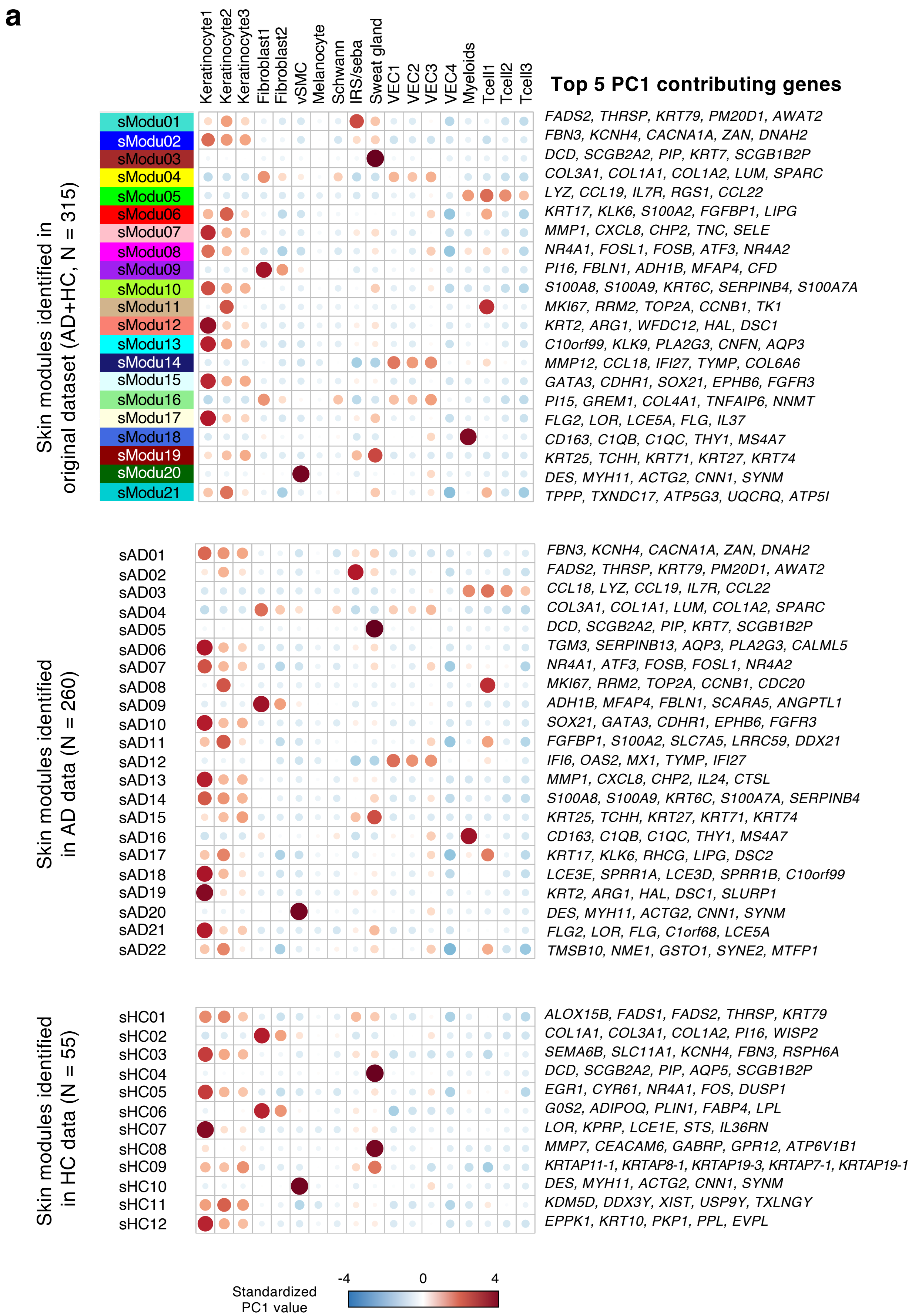

a
Keratinocyte1
Keratinocyte2
Keratinocyte3
Fibroblast1
Fibroblast2
vSMC
Melanocyte
Schwann
IRS/seba
Sweat gland
VEC1
VEC2
VEC3
VEC4
Myeloids
Tcell1
Tcell2
Tcell3
Top 5 PC1 contributing genes
Skin modules identified in original dataset (AD+HC, N = 315)
sModu01 FADS2, THRSP, KRT79, PM20D1, AWAT2
sModu02 FBN3, KCNH4, CACNA1A, ZAN, DNAH2
sModu03 DCD, SCGB2A2, PIP, KRT7, SCGB1B2P
sModu04 COL3A1, COL1A1, COL1A2, LUM, SPARC
sModu05 LYZ, CCL19, IL7R, RGS1, CCL22
sModu06 KRT17, KLK6, S100A2, FGFBP1, LIPG
sModu07 MMP1, CXCL8, CHP2, TNC, SELE
sModu08 NR4A1, FOSL1, FOSB, ATF3, NR4A2
sModu09 PI16, FBLN1, ADH1B, MFAP4, CFD
sModu10 S100A8, S100A9, KRT6C, SERPINB4, S100A7A
sModu11 MKI67, RRM2, TOP2A, CCNB1, TK1
sModu12 KRT2, ARG1, WFDC12, HAL, DSC1
sModu13 C10orf99, KLK9, PLA2G3, CNFN, AQP3
sModu14 MMP12, CCL18, IFI27, TYMP, COL6A6
sModu15 GATA3, CDHR1, SOX21, EPHB6, FGFR3
sModu16 PI15, GREM1, COL4A1, TNFAIP6, NNMT
sModu17 FLG2, LOR, LCE5A, FLG, IL37
sModu18 CD163, C1QB, C1QC, THY1, MS4A7
sModu19 KRT25, TCHH, KRT71, KRT27, KRT74
sModu20 DES, MYH11, ACTG2, CNN1, SYNM
sModu21 TPPP, TXNDC17, ATP5G3, UQCRQ, ATP5I
Skin modules identified in AD data (N = 260)
sAD01 FBN3, KCNH4, CACNA1A, ZAN, DNAH2
sAD02 FADS2, THRSP, KRT79, PM20D1, AWAT2
sAD03 CCL18, LYZ, CCL19, IL7R, CCL22
sAD04 COL3A1, COL1A1, LUM, COL1A2, SPARC
sAD05 DCD, SCGB2A2, PIP, KRT7, SCGB1B2P
sAD06 TGM3, SERPINB13, AQP3, PLA2G3, CALML5
sAD07 NR4A1, ATF3, FOSB, FOSL1, NR4A2
sAD08 MKI67, RRM2, TOP2A, CCNB1, CDC20
sAD09 ADH1B, MFAP4, FBLN1, SCARA5, ANGPTL1
sAD10 SOX21, GATA3, CDHR1, EPHB6, FGFR3
sAD11 FGFBP1, S100A2, SLC7A5, LRRC59, DDX21
sAD12 IFI6, OAS2, MX1, TYMP, IFI27
sAD13 MMP1, CXCL8, CHP2, IL24, CTSL
sAD14 S100A8, S100A9, KRT6C, S100A7A, SERPINB4
sAD15 KRT25, TCHH, KRT27, KRT71, KRT74
sAD16 CD163, C1QB, C1QC, THY1, MS4A7
sAD17 KRT17, KLK6, RHCG, LIPG, DSC2
sAD18 LCE3E, SPRR1A, LCE3D, SPRR1B, C10orf99
sAD19 KRT2, ARG1, HAL, DSC1, SLURP1
sAD20 DES, MYH11, ACTG2, CNN1, SYNM
sAD21 FLG2, LOR, FLG, C1orf68, LCE5A
sAD22 TMSB10, NME1, GSTO1, SYNE2, MTFP1
Skin modules identified in HC data (N = 55)
sHC01 ALOX15B, FADS1, FADS2, THRSP, KRT79
sHC02 COL1A1, COL3A1, COL1A2, PI16, WISP2
sHC03 SEMA6B, SLC11A1, KCNH4, FBN3, RSPH6A
sHC04 DCD, SCGB2A2, PIP, AQP5, SCGB1B2P
sHC05 EGR1, CYR61, NR4A1, FOS, DUSP1
sHC06 G0S2, ADIPOQ, PLIN1, FABP4, LPL
sHC07 LOR, KPRP, LCE1E, STS, IL36RN
sHC08 MMP7, CEACAM6, GABRP, GPR12, ATP6V1B1
sHC09 KRTAP11-1, KRTAP8-1, KRTAP19-3, KRTAP7-1, KRTAP19-1
sHC10 DES, MYH11, ACTG2, CNN1, SYNM
sHC11 KDM5D, DDX3Y, XIST, USP9Y, TXLNGY
sHC12 EPPK1, KRT10, PKP1, PPL, EVPL
Standardized PC1 value
-4
0
4

b

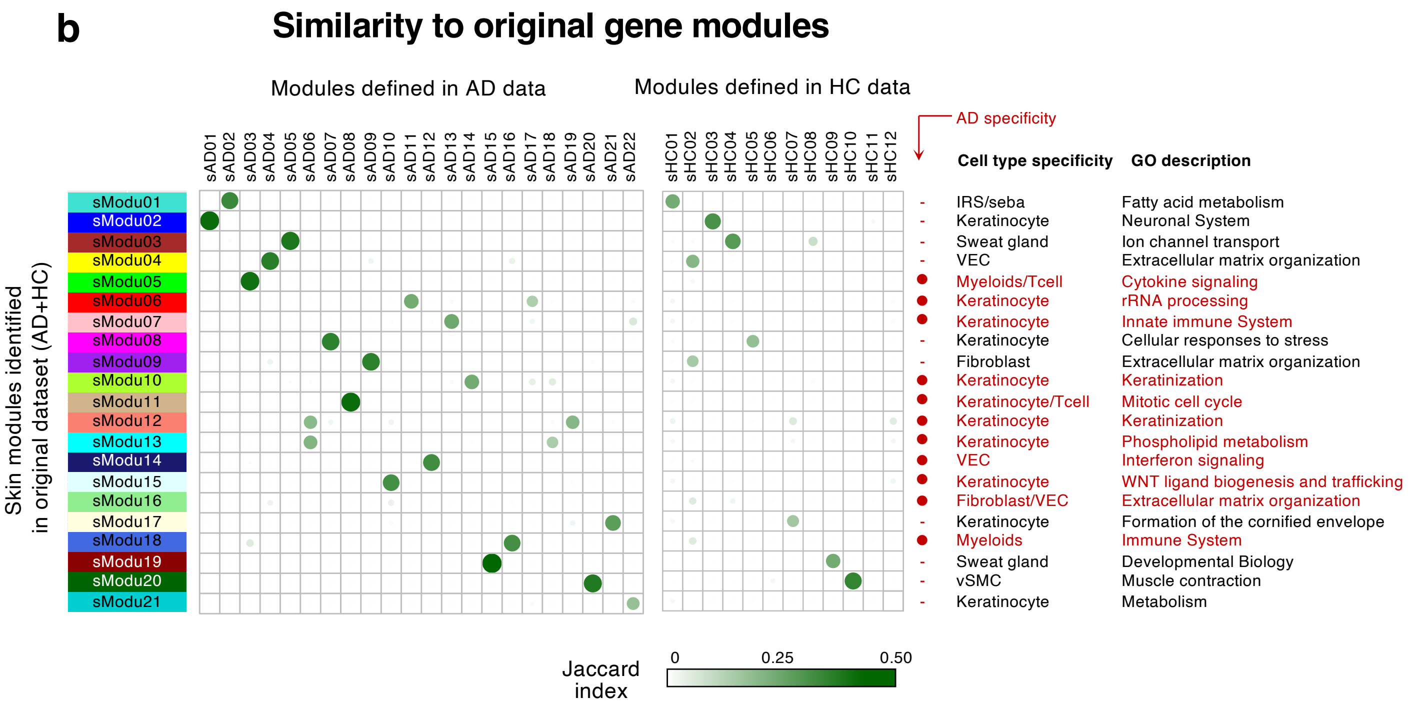
Similarity to original gene modules
Modules defined in AD data
Modules defined in HC data
AD specificity
Cell type specificity
GO description
Skin modules identified in original dataset (AD+HC)
sAD01 sAD02 sAD03 sAD04 sAD05 sAD06 sAD07 sAD08 sAD09 sAD10 sAD11 sAD12 sAD13 sAD14 sAD15 sAD16 sAD17 sAD18 sAD19 sAD20 sAD21 sAD22
sHC01 sHC02 sHC03 sHC04 sHC05 sHC06 sHC07 sHC08 sHC09 sHC10 sHC11 sHC12
sModu01 - IRS/seba - Fatty acid metabolism
sModu02 - Keratinocyte - Neuronal System
sModu03 - Sweat gland - Ion channel transport
sModu04 - VEC - Extracellular matrix organization
sModu05 - Myeloids/Tcell - Cytokine signaling
sModu06 - Keratinocyte - rRNA processing
sModu07 - Keratinocyte - Innate immune System
sModu08 - Keratinocyte - Cellular responses to stress
sModu09 - Fibroblast - Extracellular matrix organization
sModu10 - Keratinocyte - Keratinization
sModu11 - Keratinocyte/Tcell - Mitotic cell cycle
sModu12 - Keratinocyte - Keratinization
sModu13 - Keratinocyte - Phospholipid metabolism
sModu14 - VEC - Interferon signaling
sModu15 - Keratinocyte - WNT ligand biogenesis and trafficking
sModu16 - Fibroblast/VEC - Extracellular matrix organization
sModu17 - Keratinocyte - Formation of the cornified envelope
sModu18 - Myeloids - Immune System
sModu19 - Sweat gland - Developmental Biology
sModu20 - vSMC - Muscle contraction
sModu21 - Keratinocyte - Metabolism
Jaccard index
0
0.25
0.50

c

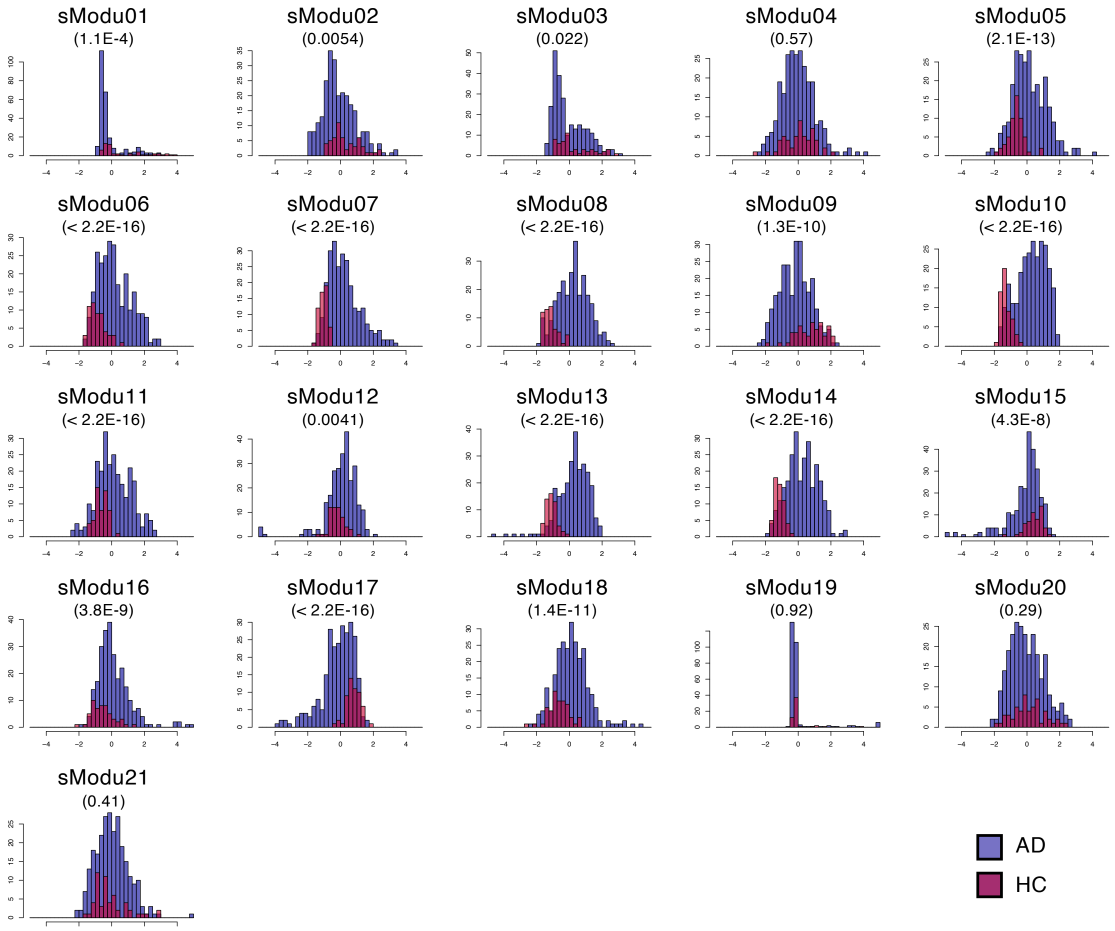
sModu01 (1.1E-4)
sModu02 (0.0054)
sModu03 (0.022)
sModu04 (0.57)
sModu05 (2.1E-13)
sModu06 (< 2.2E-16)
sModu07 (< 2.2E-16)
sModu08 (< 2.2E-16)
sModu09 (1.3E-10)
sModu10 (< 2.2E-16)
sModu11 (< 2.2E-16)
sModu12 (0.0041)
sModu13 (< 2.2E-16)
sModu14 (< 2.2E-16)
sModu15 (4.3E-8)
sModu16 (3.8E-9)
sModu17 (< 2.2E-16)
sModu18 (1.4E-11)
sModu19 (0.92)
sModu20 (0.29)
sModu21 (0.41)
AD
HC

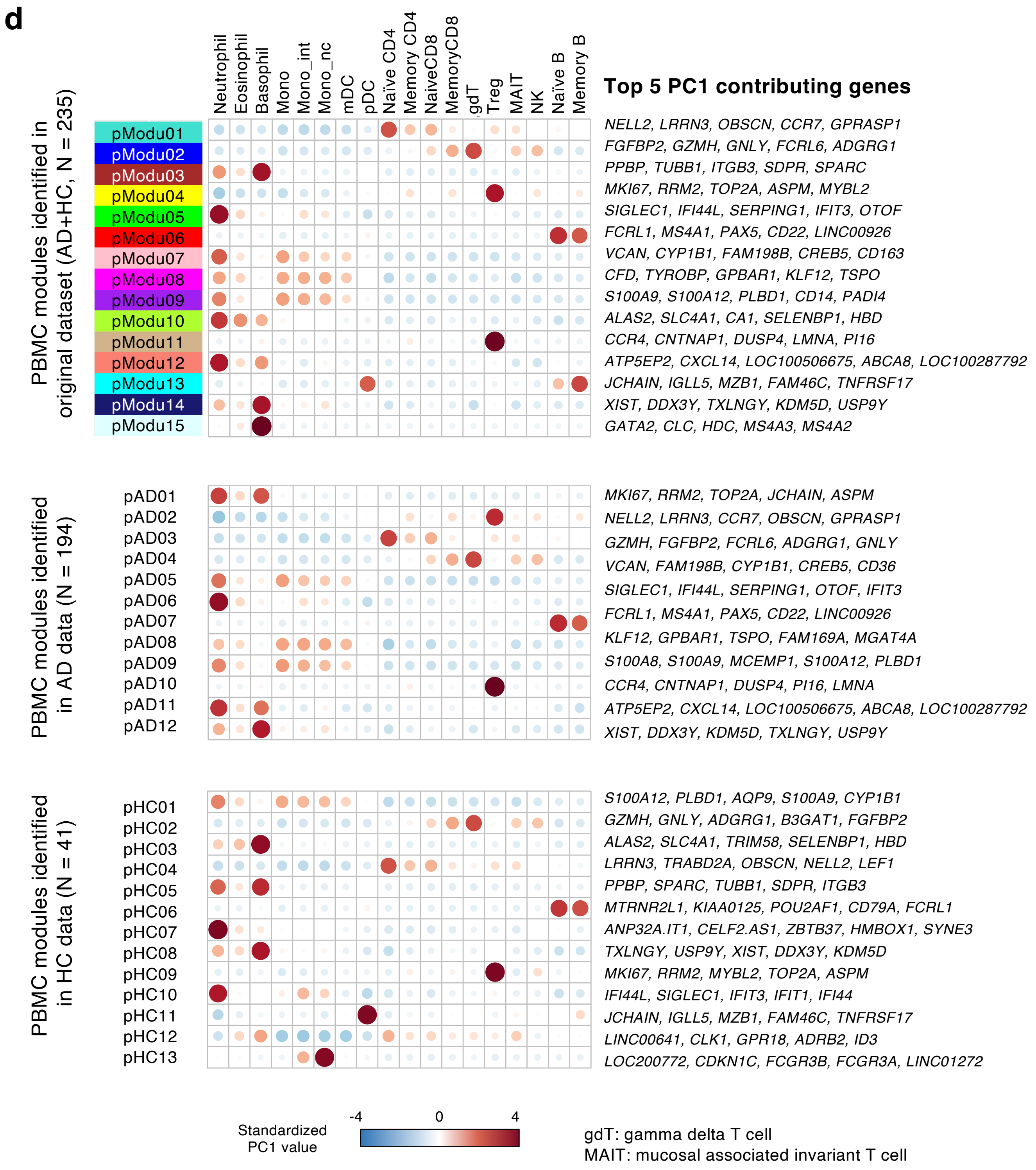
d
Neutrophil
Eosinophil
Basophil
Mono
Mono_int
Mono_nc
mDC
pDC
Naïve CD4
Memory CD4
NaiveCD8
MemoryCD8
gdT
Treg
MAIT
NK
Naïve B
Memory B
Top 5 PC1 contributing genes
PBMC modules identified in original dataset (AD+HC, N = 235)
pModu01 NELL2, LRRN3, OBSCN, CCR7, GPRASP1
pModu02 FGFBP2, GZMH, GNLY, FCRL6, ADGRG1
pModu03 PPBP, TUBB1, ITGB3, SDPR, SPARC
pModu04 MKI67, RRM2, TOP2A, ASPM, MYBL2
pModu05 SIGLEC1, IFI44L, SERPING1, IFIT3, OTOF
pModu06 FCRL1, MS4A1, PAX5, CD22, LINC00926
pModu07 VCAN, CYP1B1, FAM198B, CREB5, CD163
pModu08 CFD, TYROBP, GPBAR1, KLF12, TSPO
pModu09 S100A9, S100A12, PLBD1, CD14, PADI4
pModu10 ALAS2, SLC4A1, CA1, SELENBP1, HBD
pModu11 CCR4, CNTNAP1, DUSP4, LMNA, PI16
pModu12 ATP5EP2, CXCL14, LOC100506675, ABCA8, LOC100287792
pModu13 JCHAIN, IGLL5, MZB1, FAM46C, TNFRSF17
pModu14 XIST, DDX3Y, TXLNGY, KDM5D, USP9Y
pModu15 GATA2, CLC, HDC, MS4A3, MS4A2
PBMC modules identified in AD data (N = 194)
pAD01 MKI67, RRM2, TOP2A, JCHAIN, ASPM
pAD02 NELL2, LRRN3, CCR7, OBSCN, GPRASP1
pAD03 GZMH, FGFBP2, FCRL6, ADGRG1, GNLY
pAD04 VCAN, FAM198B, CYP1B1, CREB5, CD36
pAD05 SIGLEC1, IFI44L, SERPING1, OTOF, IFIT3
pAD06 FCRL1, MS4A1, PAX5, CD22, LINC00926
pAD07 KLF12, GPBAR1, TSPO, FAM169A, MGAT4A
pAD08 S100A8, S100A9, MCEMP1, S100A12, PLBD1
pAD09 CCR4, CNTNAP1, DUSP4, PI16, LMNA
pAD10 ATP5EP2, CXCL14, LOC100506675, ABCA8, LOC100287792
pAD11 XIST, DDX3Y, KDM5D, TXLNGY, USP9Y
pAD12
PBMC modules identified in HC data (N = 41)
pHC01 S100A12, PLBD1, AQP9, S100A9, CYP1B1
pHC02 GZMH, GNLY, ADGRG1, B3GAT1, FGFBP2
pHC03 ALAS2, SLC4A1, TRIM58, SELENBP1, HBD
pHC04 LRRN3, TRABD2A, OBSCN, NELL2, LEF1
pHC05 PPBP, SPARC, TUBB1, SDPR, ITGB3
pHC06 MTRNR2L1, KIAA0125, POU2AF1, CD79A, FCRL1
pHC07 ANP32A.IT1, CELF2.AS1, ZBTB37, HMBOX1, SYNE3
pHC08 TXLNGY, USP9Y, XIST, DDX3Y, KDM5D
pHC09 MKI67, RRM2, MYBL2, TOP2A, ASPM
pHC10 IFI44L, SIGLEC1, IFIT3, IFIT1, IFI44
pHC11 JCHAIN, IGLL5, MZB1, FAM46C, TNFRSF17
pHC12 LINC00641, CLK1, GPR18, ADRB2, ID3
pHC13 LOC200772, CDKN1C, FCGR3B, FCGR3A, LINC01272
Standardized PC1 value
-4
0
4
gdT: gamma delta T cell
MAIT: mucosal associated invariant T cell

e

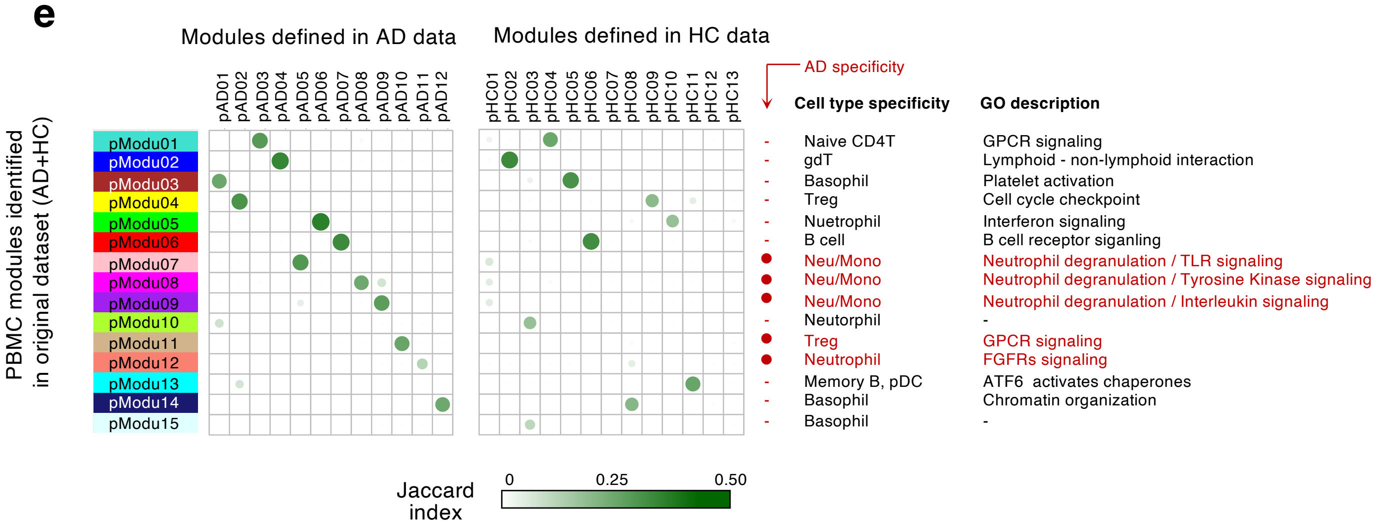

f

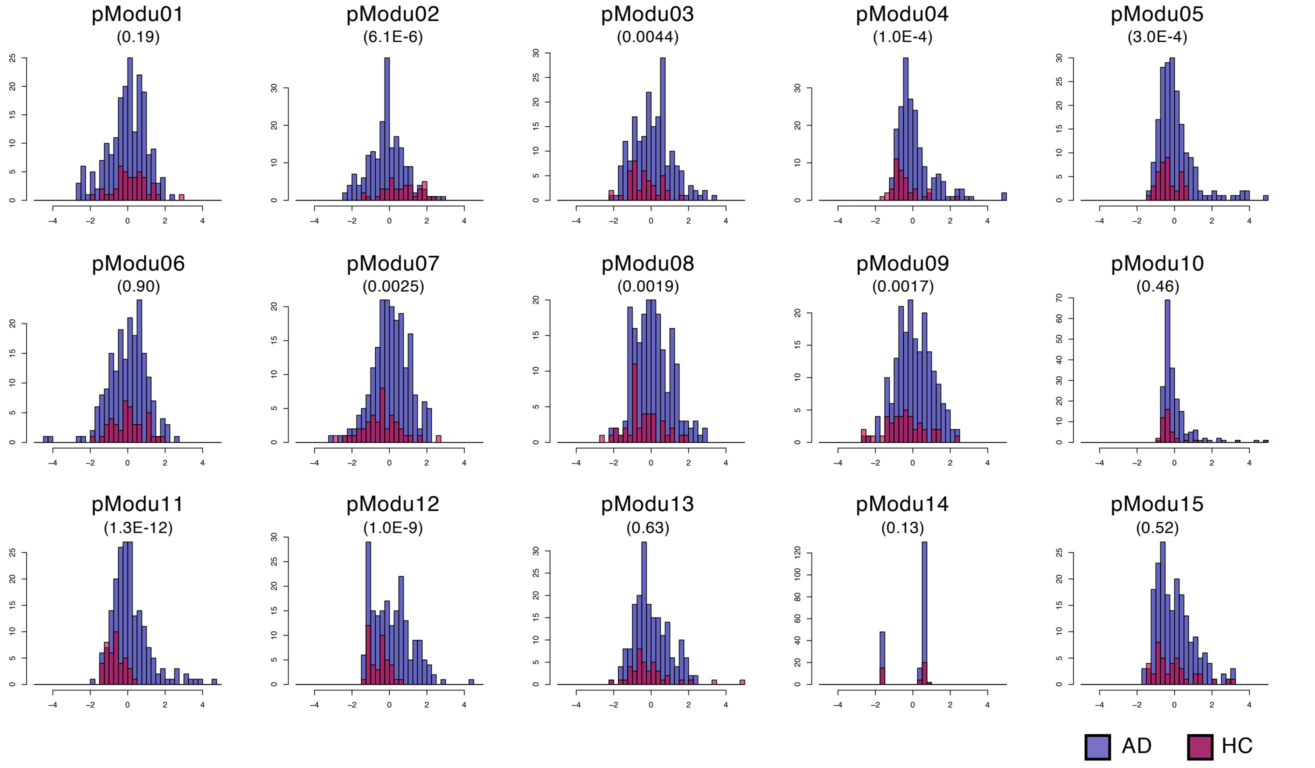

Supplementary Figure 10 Application of WGCNA on the AD/HC subset of data highlight differential patterns of AD-specific modules and generally observed modules in the tissue.

a,d. Cell type specific expression (left) and the list of top 5 PC1 contributing genes (right) of skin (**a**) and PBMC (**d**) modules identified in original dataset (upper), subset of data from AD patients (middle), and subset of data from HCs (lower). Cell type specificity was assessed by referring external dataset of skin single cell-RNA-seq (**a**) and sorted blood cell type RNA-seq data (**d**). **b,e.** Plots showing similarity between the modules defined in original dataset and modules defined in AD or HC dataset as evaluated by Jaccard index in skin (**b**) and PBMC (**e**). **c,f.** Histogram showing distribution of expression intensity of modules in AD patients (blue) and HCs (red) in skin (**c**) and PBMC (**f**). *P*-values of two-sided student's t-test or two-sided Welch's t-test (according to the homoscedasticity examined by F-test) between AD patients and HCs were described inside the brackets. Source data are provided as a Source Data file. AD: atopic dermatitis, HC: healthy control.

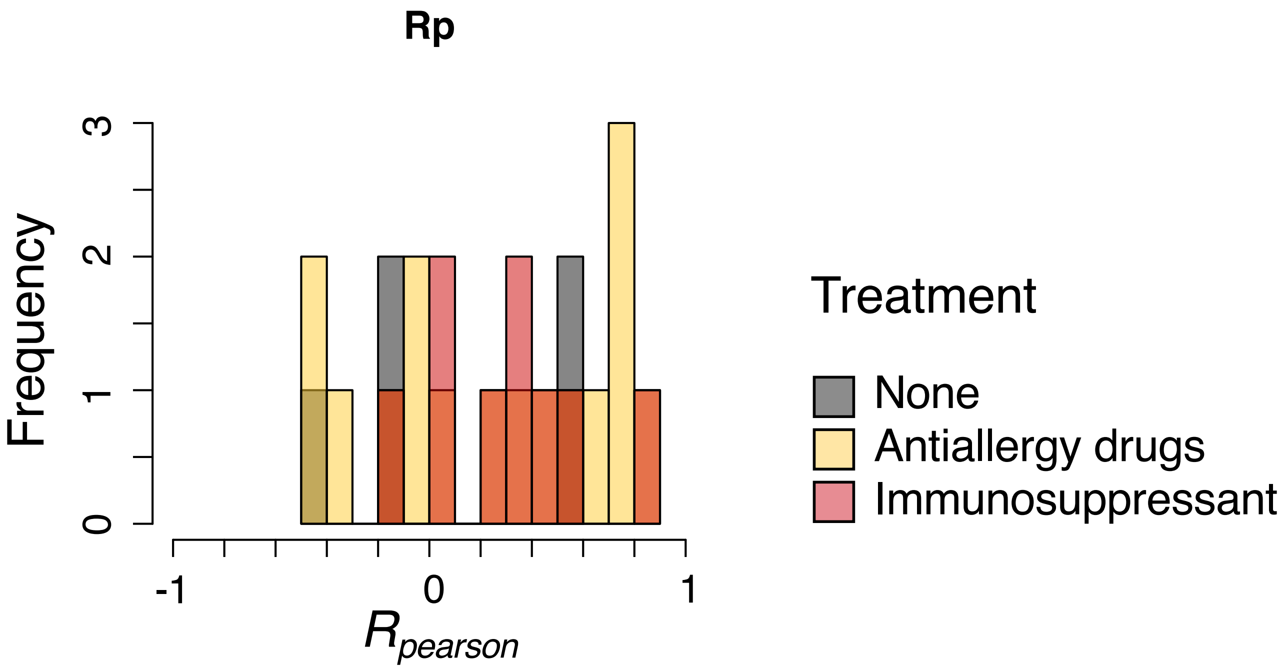

Supplementary Figure 11 Prediction accuracy of personalized disease course.
The histogram indicates the distribution of Pearson correlation coefficient between observed EASI and predicted EASI in intra-patient dynamics. There was no significant difference in the size of the coefficient among patients regarding treatment classes (Kruskal-Wallis rank sum test, p = 0.57). Source data are provided as a Source Data file.

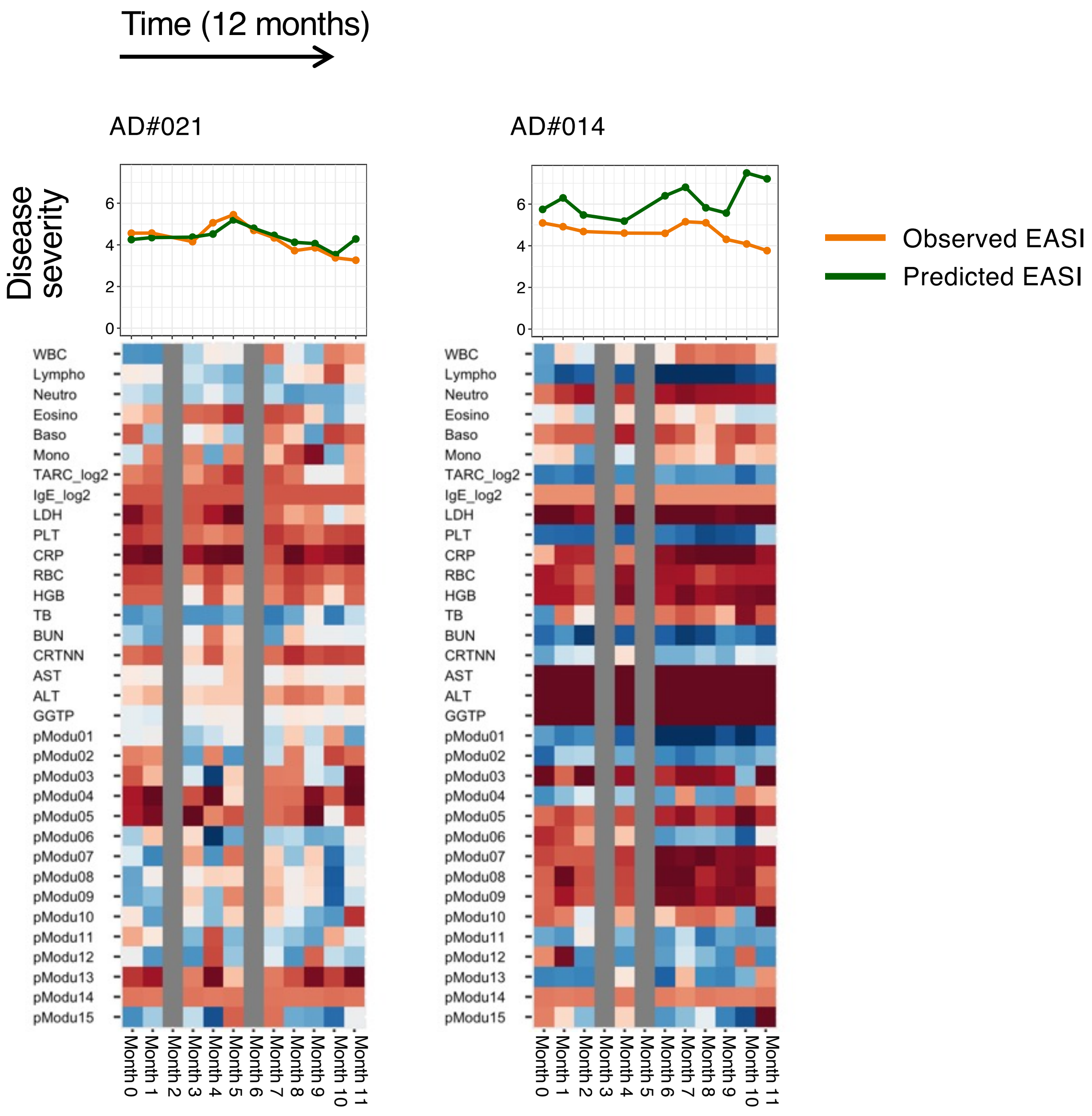

Supplementary Figure 12 Association of omics features with clinical severity in a longitudinal setting. Trajectories of observed/predicted EASI (upper) and intensity of blood examination and PBMC transcriptome modules (lower) in one year in two representative patients.

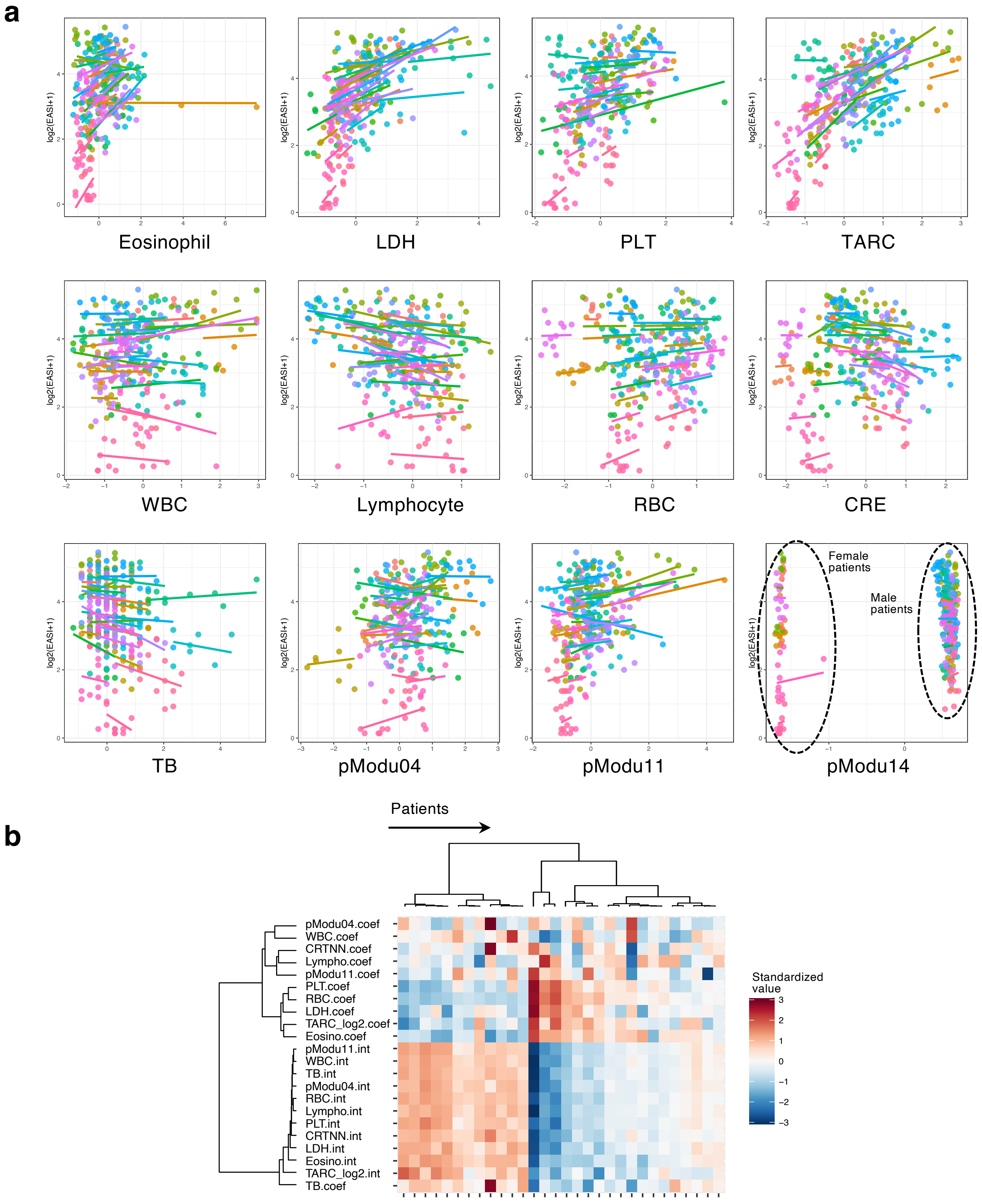

Supplementary Figure 13 Application of linear mixed model on time series data highlighted the varying random effects by patients.

a. Respective blood derived parameter was tested for linear relationship with disease severity, with patient IDs used as random effects. The data was plot with different colors according to the patients. The lines represent predicted values in each patient using the LMM with random effects. **b.** The size of random effects of both intercept and coefficient in individual patients. Selected parameters with fixed effect coefficient *p*-value (not adjusted) < 0.05 and/or random effect coefficient SD > 0.13 were shown. coef: coefficient, int: intersect.

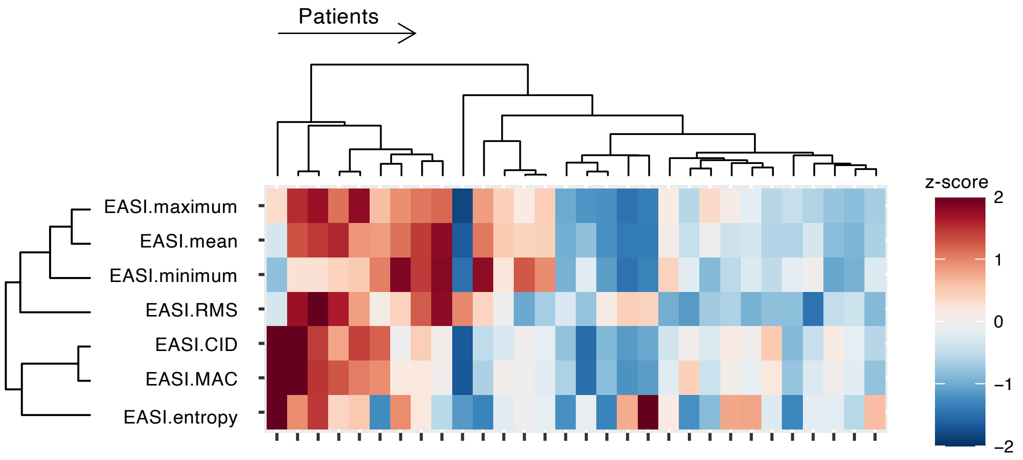

Supplementary Figure 14 Time series features in 1-year monitoring of 30 AD patients.
Hierarchical clustering of 7 types of time series features of clinical severity. RMS: root mean square, MAC: mean absolute change, CID: complexity-invariant distance.

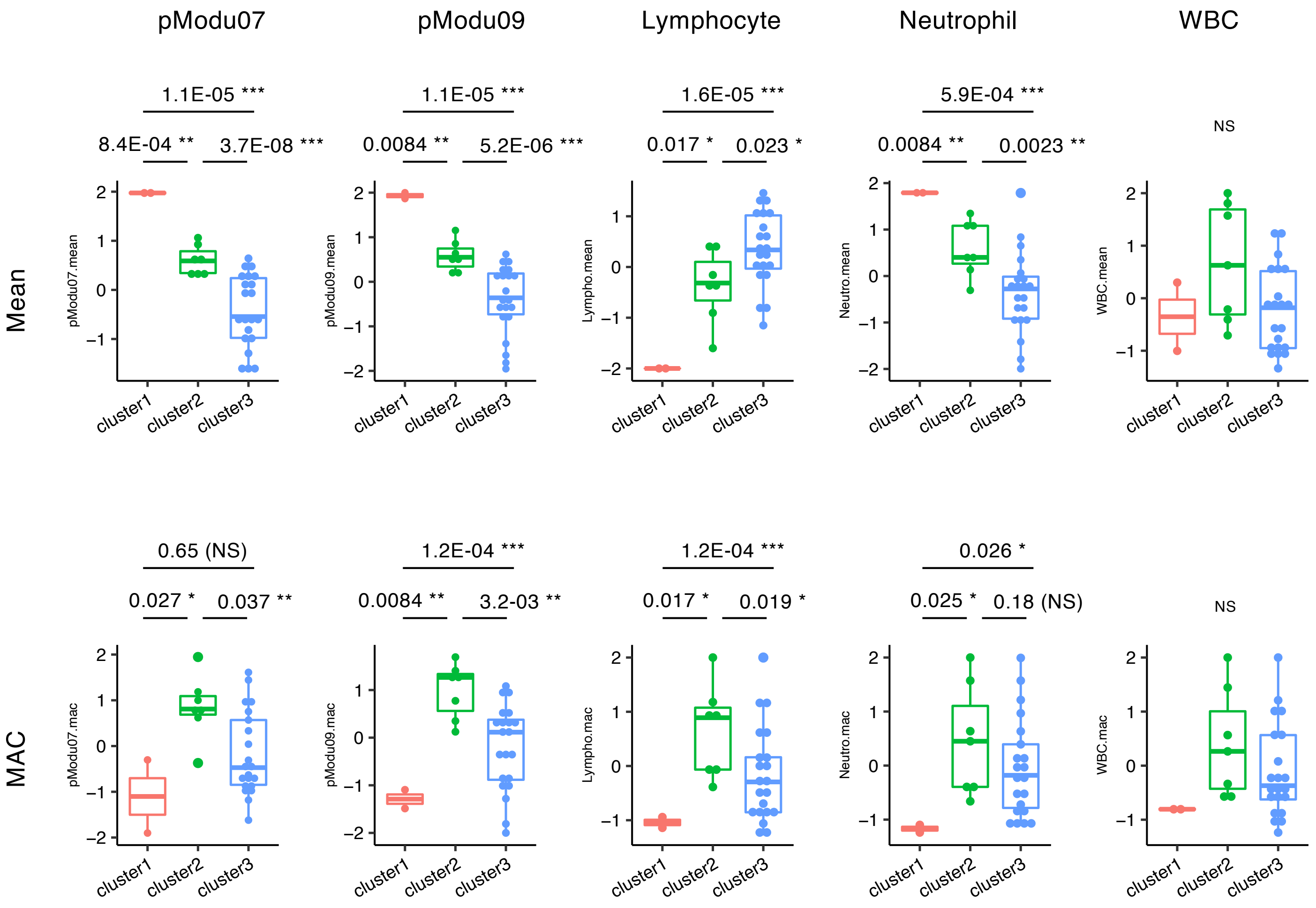

Supplementary Figure 15 Comparison of time series features in top contributing factors among three patient clusters in AD.

Two classes of time series features: mean (upper) and mean absolute change (MAC, lower) of top contributing factors in principal component analysis of time series features across patients. Boxplots show median and first and third quartiles, whiskers extending to the highest and lowest values no further than 1.5*interquartile range. Brunner-Munzel rank test. Multiple comparison tests were carried out using Kruskal-Wallis test. *P*-values less than 0.05 were considered as significant and subsequently tested for post-hoc comparison with Brunner-Munzel test with *p*-value correction by Holm's method. NS: not significant, *$p < 0.05$, **$p < 0.01$, ***$p < 0.001$. N; cluster1 = 2, cluster2 = 7, cluster3 = 21 (biologically independent samples). Source data are provided as a Source Data file.

Supplementary Tables

Supplementary Table 1 Pilosebaceous unit-related gene set defined for filtering skin samples. Pilosebaceous unit-related genes were extracted by comparison between two patient clusters identified by applying unsupervised k-means clustering (k = 2) on healthy controls.

Gene
ALOX15B
ADGRL3
FABP7
THRSP
ACSBG1
CYP4F8
SEC14L6
FADS1
FAR2
SOAT1
CRAT
AWAT1
MGST1
CIDEA
ELOVL5
INSIG1
PNPLA5
APOC1
TMEM56
PM20D1
ELOVL3
KRT79
FADS2
GAL
AWAT2
AADACL3
DGAT2L6

Supplementary Table 2 Drugs used for systemic treatment of AD in this study population.

Drug name	Category
Cyclospoin A	Immunosuppressant
Bepotastine	Antiallergic drug
Bilrastine	Antiallergic drug
Clemastine Fumarate	Antiallergic drug
d-Chlorpheniramine Maleate	Antiallergic drug
Desloratadine	Antiallergic drug
Epinastine	Antiallergic drug
Fexofenadine	Antiallergic drug
Hydroxyzine	Antiallergic drug
Levocetirizine	Antiallergic drug
Olopatadine	Antiallergic drug
Rupatadine fumarate	Antiallergic drug
Suplatast tosylate	Antiallergic drug

Supplementary Table 3 Characteristics of study participants.

		Cross-sectional		Longitudinal
		AD	Healthy control	AD
General information	Number of participants	115	14	30
	Sex	Male 85<br>Female 30	Male 9<br>Female 5	Male 23<br>Female 7
	Age (mean ± SD)	40.5 ± 11.6	47.3 ± 10.7	36.4 ± 10.0
	EASI (mean ± SD)	15.6 ± 11.6	-	14.0 ± 7.96
Treatment	Topical steroids	110	-	30
	Oral immunosuppressant	4	-	9
	Oral steroids	2	-	0
	Oral antiallergic drugs	26	-	Constant: 12<br>Occasional: 2

Supplementary Table 4 Combination of cytokine genes and receptor genes frequently observed in each participant group.

Disease	Cytokine	Receptor	cytokine_type	receptor_type	#Connection	#Connection/ #Patients
AD	CCL22	CCR4	Skin	Skin	40	0.348
AD	CCL17	CCR4	Skin	Skin	39	0.339
AD	CCL13	CCR1	Skin	Skin	38	0.330
AD	CCL18	CCR8	Skin	Skin	38	0.330
AD	CCL19	CCR7	Skin	Skin	38	0.330
AD	IL13	IL4R	Skin	Skin	38	0.330
AD	IL13	IL13RA2	Skin	Skin	36	0.313
AD	IL36A	IL1RL2	Skin	Skin	36	0.313
AD	CXCL8	CXCR1	Skin	Skin	35	0.304
AD	CCL13	ACKR4	Skin	Skin	34	0.296
AD	CCL13	CCR2	Skin	Skin	34	0.296
AD	CCL2	CCR2	Skin	Skin	34	0.296
AD	CXCL1	CXCR1	Skin	Skin	34	0.296
AD	IL36G	IL1RL2	Skin	Skin	34	0.296
AD	IL36RN	IL1RL2	Skin	Skin	34	0.296
AD	CCL18	CCR8	Skin	PBMC	33	0.287
AD	CCL2	CCR4	Skin	Skin	33	0.287
AD	CCL2	ACKR4	Skin	Skin	32	0.278
AD	IL20	IL20RB	Skin	PBMC	30	0.261
AD	CCL19	ACKR4	Skin	Skin	28	0.243
AD	IL13	IL4R	Skin	PBMC	28	0.243
AD	IL6	IL6R	Skin	Skin	28	0.243
AD	CXCL10	CXCR3	Skin	Skin	27	0.235
AD	IL20	IL20RB	Skin	Skin	27	0.235
AD	IL36G	IL1RAP	Skin	PBMC	27	0.235
AD	CCL1	CCR8	Skin	Skin	26	0.226
AD	CCL13	ACKR1	Skin	Skin	26	0.226
AD	CCL13	CCR3	Skin	Skin	26	0.226
AD	CCL17	CCR4	Skin	PBMC	26	0.226
AD	CCL22	CCR4	Skin	PBMC	26	0.226
AD	FPR3	ANXA1	Skin	Skin	26	0.226
AD	IL13	IL13RA1	Skin	Skin	26	0.226
AD	IL36A	IL1RAP	Skin	PBMC	26	0.226
AD	PPBP	CXCR2	Skin	Skin	26	0.226
AD	CCL13	CCR1	Skin	PBMC	25	0.217
AD	CCL13	CCR3	Skin	PBMC	25	0.217
AD	CCL2	CCR2	Skin	PBMC	25	0.217
AD	CCL2	CCR4	Skin	PBMC	25	0.217
AD	CCL26	CCR3	Skin	Skin	25	0.217
AD	IL15	IL2RG	Skin	Skin	25	0.217
AD	IL36RN	IL1RAP	Skin	Skin	25	0.217
AD	IL6	IL6ST	Skin	Skin	25	0.217
AD	CXCL8	CXCR1	PBMC	PBMC	25	0.217
AD	CCL1	CCR8	Skin	PBMC	24	0.209
AD	CCL17	ACKR1	Skin	Skin	24	0.209
AD	CCL2	ACKR1	Skin	Skin	24	0.209
AD	CD40LG	CD40	Skin	Skin	24	0.209
AD	IL15	IL15RA	Skin	Skin	24	0.209
AD	IL36G	IL1RAP	Skin	Skin	24	0.209
AD	IL36RN	IL1RAP	Skin	PBMC	24	0.209
AD	IL6	IL6ST	Skin	PBMC	24	0.209
AD	CXCL14	CXCR4	PBMC	PBMC	24	0.209
AD	CXCL8	CXCR1	PBMC	Skin	24	0.209
AD	CXCL8	CXCR2	PBMC	PBMC	24	0.209
AD	CCL13	CCR2	Skin	PBMC	23	0.200
AD	CCL21	CCR7	Skin	Skin	23	0.200
AD	IL20	IL22RA1	Skin	Skin	23	0.200
AD	OSM	OSMR	Skin	Skin	23	0.200

Disease	Cytokine	Receptor	cytokine_type	receptor_type	#Connection	#Connection/ #Patients
normal	IL37	IL18R1	Skin	PBMC	13	0.929
normal	IL34	CSF1R	Skin	Skin	12	0.857
normal	IL37	IL18RAP	Skin	PBMC	11	0.786
normal	IL18	IL18R1	Skin	PBMC	8	0.571
normal	IL37	IL18R1	Skin	Skin	8	0.571
normal	CCL5	CCR5	PBMC	PBMC	7	0.500
normal	IL18	IL18RAP	Skin	PBMC	6	0.429
normal	IL1A	IL1R1	Skin	Skin	6	0.429
normal	IL37	IL18RAP	Skin	Skin	6	0.429
normal	CCL5	ACKR1	PBMC	Skin	6	0.429
normal	IL16	CD4	Skin	Skin	5	0.357
normal	IL18	IL18R1	Skin	Skin	5	0.357
normal	IL1A	IL1R2	Skin	PBMC	5	0.357
normal	IL1F10	IL1RL2	Skin	Skin	5	0.357
normal	CCL5	CCR3	PBMC	Skin	5	0.357
normal	CCL28	CCR3	Skin	Skin	4	0.286
normal	IL1A	IL1R2	Skin	Skin	4	0.286
normal	IL1A	IL1R1	Skin	PBMC	4	0.286
normal	LIF	IL6ST	Skin	Skin	4	0.286
normal	CCL4L1	CCR5	PBMC	PBMC	4	0.286
normal	CXCL5	CXCR1	PBMC	PBMC	4	0.286
normal	XCL2	XCR1	PBMC	Skin	4	0.286
normal	BMP5	BMPR1B	Skin	Skin	3	0.214
normal	BMP5	BMPR1B	Skin	PBMC	3	0.214
normal	BMP6	ACVR2A	Skin	Skin	3	0.214
normal	BMP6	BMPR2	Skin	Skin	3	0.214
normal	CCL21	ACKR4	Skin	Skin	3	0.214
normal	CCL21	CCR7	Skin	PBMC	3	0.214
normal	CXCL5	CXCR1	Skin	PBMC	3	0.214
normal	CXCL5	CXCR2	Skin	PBMC	3	0.214
normal	EPO	EPOR	Skin	Skin	3	0.214
normal	IL17C	IL17RE	Skin	PBMC	3	0.214
normal	IL18	IL18RAP	Skin	Skin	3	0.214
normal	IL1A	IL1RAP	Skin	Skin	3	0.214
normal	IL1F10	IL1RAP	Skin	PBMC	3	0.214
normal	IL24	IL20RB	Skin	PBMC	3	0.214
normal	IL33	IL1RL1	Skin	Skin	3	0.214
normal	IL33	IL1RAP	Skin	PBMC	3	0.214
normal	IL34	CSF1R	Skin	PBMC	3	0.214
normal	BMP6	ACVR2A	PBMC	Skin	3	0.214
normal	BMP6	BMPR1A	PBMC	Skin	3	0.214
normal	CXCL5	CXCR2	PBMC	PBMC	3	0.214
normal	PPBP	CXCR2	PBMC	PBMC	3	0.214
normal	XCL1	XCR1	Skin	PBMC	3	0.214

Supplementary Table 5 Summary of cell types highly involved in cytokine-receptor coupling in each participant group.

	Cytokine			Receptor		
	Tissue	Cell type	#Connection	Tissue	Cell type	#Connection
AD	Skin	Tcell	119	PBMC	non-specific	116
	Skin	VEC	90	Skin	Myeloids	87
	PBMC	non-specific	58	Skin	Tcell	69
	Skin	Myeloids	57	Skin	VEC	57
	PBMC	Mono	56	PBMC	Mono	39
	Skin	KC	56	Skin	FB	37
	Skin	non-specific	46	Skin	non-specific	34
	Skin	vSMC	23	PBMC	Treg	25
	Skin	FB	18	Skin	KC	20
	PBMC	DC_mye	16	PBMC	DC_mye	18
	PBMC	NK	14	PBMC	CD4T	17
	PBMC	CD8T	13	PBMC	T_MAIT	16
	Skin	Sweat	9	PBMC	NK	14
Healthy control	Skin	Tcell	33	PBMC	non-specific	44
	Skin	VEC	29	Skin	Myeloids	35
	PBMC	non-specific	27	Skin	VEC	19
	Skin	non-specific	27	Skin	Tcell	17
	Skin	KC	21	PBMC	Mono	12
	Skin	Myeloids	21	Skin	FB	11
	PBMC	Mono	18	Skin	non-specific	11
	PBMC	CD8T	8	PBMC	DC_mye	10
	Skin	FB	8	Skin	KC	10
	PBMC	DC_mye	7	PBMC	Treg	8
	Skin	Sweat	6	Skin	Melano	8
	PBMC	NK	5	PBMC	DC_pls	7
	Skin	vSMC	5	PBMC	T_MAIT	7

Supplementary Table 6 A list of blood test variables used in regression models.
Individual variables were standardized across study subjects. WBC: white blood cell, RBC: red blood cell, LDH: lactate dehydrogenase, PLT: platelet, CRP: C-reactive protein, HGB: hemoglobin, TB: total bilirubin, BUN: blood urea nitrogen, CRE: creatinine, AST: aspartate aminotransferase, ALT: alanine transaminase, GGTP: gamma-glutamyl transferase.

Category	Variable	Unit	Mean	±	SD
Hemogram	WBC	10^3 cell/µL	6.73	±	1.73
Hemogram	RBC	10^6 cell/µL	4.86	±	0.51
Hemogram	Lymphocytes	%	24.41	±	7.84
Hemogram	Neutrophil	%	62.71	±	8.33
Hemogram	Eosinophil	%	6.01	±	4.71
Hemogram	Basophil	%	0.68	±	0.37
Hemogram	Monocyte	%	6.09	±	1.74
Biochemistry	LDH	U/L	235.50	±	75.14
Biochemistry	PLT	10^3 cell/µL	271.96	±	57.31
Biochemistry	CRP	mg/dL	0.16	±	0.36
Biochemistry	HGB	g/dL	14.52	±	1.59
Biochemistry	TB	mg/dL	0.71	±	0.34
Biochemistry	BUN	mg/dL	13.69	±	2.83
Biochemistry	CRE	mg/dL	0.80	±	0.15
Biochemistry	AST	U/L	23.06	±	8.72
Biochemistry	ALT	U/L	26.69	±	21.18
Biochemistry	GGTP	U/L	29.26	±	20.08
Immunoassay	Total IgE (log2)	-	10.20	±	3.17
	(Total IgE (raw)	IU/mL	4903	±	6710)
Immunoassay	TARC (log2)	-	10.02	±	1.64
	(TARC (raw)	pg/mL	2348	±	6009)

Supplementary Table 7 Prediction variables extracted by regression analysis applied on the subset of the patients who are under treatment only with topical steroids but no internal medicine.

Elastic net regression was applied to data including basic information, blood test, skin transcriptome modules and PBMC transcriptome modules. Adjustment was made for R^2 in training set with the number of prediction variables. N; AD = 104, healthy control = 14. Variable with $p < 0.1$ are listed. sModu: skin transcriptome module, pModu: PBMC transcriptome module, ALT: alanine transaminase, BUN: blood urea nitrogen, CRP: C-reactive protein, VEC: vascular endothelial cells.

Objective variables	Adjusted R^2	Predictors	Coefficient	*P*-value	Tissue	PC1 top 5 genes	Cell type specificity
EASI (total)	Training 0.63 Test 0.51	Lymphocyte	-0.34	0.0038	Blood	-	Lymphocyte
		sModu14	0.35	0.024	Skin	*MMP12, CCL18, IFI27, TYMP, COL6A6*	VEC
		sModu10	0.29	0.071	Skin	*S100A8, S100A9, KRT6C, SERPINB4, S100A7*	Keratinocyte
		Eosinophil	0.19	0.081	Blood	-	Eosinophil
EASI (erythema)	Training 0.67 Test 0.56	sModu08	0.17	0.027	Skin	*NR4A1, FOSL1, FOSB, ATF3, NR4A2*	Keratinocyte
		TARC	0.23	0.041	Blood	-	-
		pModu11	0.13	0.069	Blood	*CCR4, CNTNAP1, DUSP4, LMNA, PI16*	Treg
EASI (papulation)	Training 0.54 Test 0.30	Lymphocyte	-0.35	0.0011	Blood	-	Lymphocyte
		sModu14	0.46	0.0019	Skin	*MMP12, CCL18, IFI27, TYMP, COL6A6*	VEC
		Eosinophil	0.22	0.0066	Blood	-	Eosinophil
		pModu06	-0.22	0.029	Blood	*FCRL1, MS4A1, PAX5, CD22, LINC00926*	B cell
		pModu01	0.22	0.040	Blood	*NELL2, LRRN3, OBSCN, CCR7, GRASP1*	Naïve CD4
		pModu03	-0.16	0.052	Blood	*PPBP, TUBB1, ITGB3, SDPR, SPARC*	Basophil
		ALT	0.15	0.055	Blood	-	-
		BUN	-0.15	0.056	Blood	-	-
		CRP	0.16	0.062	Blood	-	-
		sModu05	-0.23	0.070	Skin	*LYZ, CCL19, IL7R, RGS1, CCL22*	T cell/myeloid
		sModu16	0.22	0.075	Skin	*PI15, GREM1, COL4A1, TNFAIP6, NNMT*	VEC

Supplementary Table 8 Antibodies used for immunohistochemistry.

Antibody	Manufacturer	Catalog#	Species	Clone	Isotype	Dilution
CD4	Novus biologicals	NBP2-52670	Rabbit	13B8.2	IgG	1/500
Myeloperoxidase	Dako	A0398	Rabbit	Polyclonal	Polyclonal	1/1000
Major basic protein	Bio-Rad	MCA5751	Mouse	BMK-13	IgG1	1/200
CD206	Novus biologicals	NB600-1415	Mouse	15-2	IgG1	1/100
CD11c	BD	550375	Mouse	B-ly6	IgG1	1/10
CD1a	Novus biologicals	NBP2-34314	Mouse	O10+C1A/711	IgG1	1/200
Keratin 16	LabVision	MS-620-P1	Mouse	LL025	IgG1	1/200
Filaggrin	GeneTex	GTX23137	Mouse	FLG01	IgG1	1/100
CD31	Novus biologicals	NB600-562	Mouse	JC/70A	IgG1k	1/100
FceR1a	Bio Academia	72-003	Mouse	CRA1	IgG2b	1/100
CD208	Immunotech	IM3448	Mouse	104.G4	IgG1	1/20
2D7	Abcam	ab155577	Mouse	2D7	IgG1	1/100
CD8	Dako	M710301-2	Mouse	C8/144B	IgG1k	1/40
Lactoferrin	eBioschience	14-6604-82	Mouse	B97	IgG1	1/100

Supplementary Notes

Assessment of the influence of treatment difference on the results of regression analysis

To examine the influence of treatment difference on the results of regression analysis, we conducted subanalysis on the patient subset excluding those who are under the specific treatments. Among the cross-sectional cohort patients, 110 patients (96%) were under treatment with topical steroids, while 2 patients (1.7%) and 4 patients (3.4%) were under oral steroids and immunosuppressant, respectively, during the study period. Accordingly, the majority of the patients (104 patients, 90.4%) were under treatment only with topical steroids and free from oral steroids or oral immunosuppressant. Therefore, we applied regression analysis on this patient subset along with healthy control (total 118 subjects: 104 AD patients and 14 healthy controls). We found that the large part of the top contributing factors in the original analysis (total 129 subjects) were selected again as contributing factors in the subanalysis (**Supplementary Table 7**), suggesting that our results of phenotype – endotype association were not biased by the potential influence of the treatment difference among patients.

Differential characterization of modules related to disease and modules related to tissue homeostasis

To confirm specificity of the identified modules in AD population, we separately applied WGCNA to the data subsets consisting of the AD patients and HCs (N; AD = 260, HCs = 55 for skin, and AD = 194, HCs = 41 for PBMC), and compared the newly defined modules with the original modules. These modules defined in two different datasets were then named sAD

and sHC for skin modules, and pAD and pHC for PBMC modules, respectively (**Supplementary Fig. 10 a, d**).

Similarity of the newly defined modules to the original modules was evaluated using Jaccard index with the following formula; Jaccard index (X, Y) = $|X \cap Y| / |X \cup Y|$, where X and Y represent the genes assigned to a given module defined in original dataset (sModu and pModu), and a given module defined in subset of data (sAD/sHC and pAD/pHC), respectively (**Supplementary Fig. 10 b, e**). Newly defined modules with Jaccard index greater than 0.20 were deemed as equivalent modules to the original modules.

In the skin data, nine modules were defined in both AD and HC data, and therefore they were considered to be general modules in skin tissue, while 10 modules were defined only in AD data, which were accordingly considered to be AD specific. The general skin modules include modules specifically expressed by keratinocyte, inner root sheath/sebaceous gland (IRS/seba) and sweat gland, suggestive of metabolic homeostasis in skin tissue. On the other hand, the AD-specific modules include those specifically expressed by Tcell and myeloids along with keratinocyte, especially those characterized by the immune-related GO terms; “Cytokine signaling”, “Innate immune system”, “Interferon signaling” and “Immune system”.

In the PBMC data, nine modules were general in both AD and HC, while five modules were found to be AD specific. The general PBMC modules consisted of modules expressed by multiple cell types of both lymphocyte and myeloids with GO terms such as “Platelet activation” and “B cell receptor signaling”. On the other hand, AD specific modules include modules that are expressed by neutrophil, monocyte and Treg with the GO terms such as “Neutrophil degranulation” and “FGFRs signaling”.

Consistently, expression intensity was significantly higher in AD compared to HCs in the AD specific modules (**Supplementary Fig. 10 c, f**). In contrast, expression intensity was greater

in the HCs than the AD patients in sModu09 (GO: Extracellular matrix organization, top genes: *PI16, FBLN1, ADH1B, MFAP4, CFD*), sModu17 (GO: Formation of the cornified envelope, top genes: *FLG2, LOR, LCE5A, FLG, IL37*) in skin, suggesting the importance of these modules for normal function of epidermal barrier and connective tissue in healthy skin.

Application of linear mixed model on time series dataset

To examine the relationship between each of the blood analytes and the disease severity in individual patients in the longitudinal settings, we applied linear mixed model (LMM) on time series data using lmer() function from the lme4 R package [1]. Respective blood derived parameter was tested for linear relationship with disease severity, with patient IDs used as random effects. While fixed effect coefficient with $p > 0.05$ was considered to be significant, random effect coefficient with SD > 0.13 was considered to be at large variance across patients, suggested to be potential stratifying factors that would highlight the differential longitudinal features among patients.

Accordingly, four blood derived parameters, TARC, Eosinophil, LDH and PLT were found to have significant fixed effects in linear relationship with disease severity (coefficient $p < 0.05$), under the assumption that individual patients harbor their own random effects both in intercept and coefficient. Above all, TARC showed the smallest *p*-value and the largest coefficient value as a fixed effect, with substantial variance in random effects of both intercept and coefficient. This suggests that TARC bears varying size in both the baseline and effects on linear relation to disease severity by patients, thereby being helpful for fundamental characterization of patients in the context of inter-individual difference as well as intra-individual variation in the longitudinal setting.

Although other analytes did not show significant fixed effects in the relationship to disease severity, some of them such as creatinine (CRE) and pModu11 showed substantial variance in the random effects of coefficient across patients. In those analytes, coefficients of relationship between disease severity were found to vary largely by patients (**Supplementary Fig. 13a**), which compromise the credibility in explanation capability of the values when data from all the patients were pooled. (Note that pModu14 which represents X- or Y-chromosome linked genes is an exception, since high level of SD was obviously produced because of outliers.) This feature of parameter can be advantageously used for patient stratification since it can highlight the differential longitudinal features among patients, and therefore, we conducted patient clustering using these parameters that have SD values of random effect coefficients above 1.3 (**Supplementary Fig. 13b**).

Accordingly, we found that patients were basically stratified into two subsets; patients who have large random effect intercept and small random effect coefficient, and patients who have small random effect intercept and large random effect coefficient. It also became apparent that the size of random effect coefficient was inversely related to the size of random effect intercept in most of the parameters, especially in the parameters that have significant fixed effects (TARC, Eosinophil, LDH and PLT) as well as RBC and pModu11. This patient clustering is in part similar to the one demonstrated in **Fig. 8c** but no clinical relevance was found so far.

References

1. Bates D, Mächler M, Bolker B, Walker S: **Fitting Linear Mixed-Effects Models Using lme4**. *J Stat Softw* 2015, **67**(1):1 - 48.
