# Supplementary material for: Multifaceted analysis of cross-tissue transcriptomes reveals phenotype–endotype associations in atopic dermatitis
Source: Nat Commun. 2023 Oct 2;14:6133. doi: 10.1038/s41467-023-41857-8 (PMC10545679; doi:10.1038/s41467-023-41857-8)
Supplement: Supplementary file 3 — Reporting Summary [file 41467_2023_41857_MOESM3_ESM.pdf]

nature portfolio

Corresponding author(s): Haruhiko Koseki

Last updated by author(s): Jul 26, 2023

Reporting Summary

Nature Portfolio wishes to improve the reproducibility of the work that we publish. This form provides structure for consistency and transparency in reporting. For further information on Nature Portfolio policies, see our Editorial Policies and the Editorial Policy Checklist.

Statistics

For all statistical analyses, confirm that the following items are present in the figure legend, table legend, main text, or Methods section.

n/a	Confirmed	
☐	☒	The exact sample size (*n*) for each experimental group/condition, given as a discrete number and unit of measurement
☐	☒	A statement on whether measurements were taken from distinct samples or whether the same sample was measured repeatedly
☐	☒	The statistical test(s) used AND whether they are one- or two-sided<br>*Only common tests should be described solely by name; describe more complex techniques in the Methods section.*
☐	☒	A description of all covariates tested
☐	☒	A description of any assumptions or corrections, such as tests of normality and adjustment for multiple comparisons
☐	☒	A full description of the statistical parameters including central tendency (e.g. means) or other basic estimates (e.g. regression coefficient) AND variation (e.g. standard deviation) or associated estimates of uncertainty (e.g. confidence intervals)
☐	☒	For null hypothesis testing, the test statistic (e.g. *F*, *t*, *r*) with confidence intervals, effect sizes, degrees of freedom and *P* value noted<br>*Give P values as exact values whenever suitable.*
☒	☐	For Bayesian analysis, information on the choice of priors and Markov chain Monte Carlo settings
☒	☐	For hierarchical and complex designs, identification of the appropriate level for tests and full reporting of outcomes
☐	☒	Estimates of effect sizes (e.g. Cohen's *d*, Pearson's *r*), indicating how they were calculated

Our web collection on statistics for biologists contains articles on many of the points above.

Software and code

Policy information about availability of computer code

Data collection	RNA-seq: Fastq files were generated on an illumina HiSeq instrument and bcl2fastq (v2.20, Illumina).
Data analysis	All analysis in this study were performed using published softwares. RNA-seq alignment to human genome (GRCh38) was conducted using STAR (v2.5.2). R (v3.6.2, unless specified otherwise) and Python (v3.7.4) were used for statistical analysis and visualization. The following packages of R or Python were used in this study: Rsubread (v2.0.1), sva (v3.35.2), DESeq2 (v1.26.0), umap (v0.2.7.0), clusterProfiler (v3.14.3), ReactomePA (v1.30.0), lawstat (v3.3), circlize (v0.4.13), wgcna (v1.70.3), igraph (v1.2.4.2), glmnet (v4.1.2), Seurat (v4.3.0, with R v4.0.2), TSfresh (v0.18.0).

For manuscripts utilizing custom algorithms or software that are central to the research but not yet described in published literature, software must be made available to editors and reviewers. We strongly encourage code deposition in a community repository (e.g. GitHub). See the Nature Portfolio guidelines for submitting code & software for further information.

Data

Policy information about availability of data

All manuscripts must include a data availability statement. This statement should provide the following information, where applicable:

- Accession codes, unique identifiers, or web links for publicly available datasets
- A description of any restrictions on data availability
- For clinical datasets or third party data, please ensure that the statement adheres to our policy

All of the sequence data generated in this study are available under controlled access at the Japanese Genotype-phenotype Archive (JGA) with accession codes JGAS000628 (https://humandbs.biosciencedbc.jp/en/hum0413-v1#JGAS000628) which can be accessed through application for hum0413 at the National Bioscience Database Center (NBDC). The reference data used in this study are available in the Gene Expression Omnibus database under accession code GSE147424 (https://www.ncbi.nlm.nih.gov/geo/query/acc.cgi?acc=GSE147424) and Human Protein Atlas database (https://www.proteinatlas.org/about/download) with the title of "RNA HPA immune cell gene data".

Human research participants

Policy information about studies involving human research participants and Sex and Gender in Research.

Reporting on sex and gender	Sex (biological attribute) of the study subjects was determined based on self-reporting.<br>94 males and 35 females were analyzed in cross-sectional analysis, and 23 males and 7 females were analyzed in longitudinal analysis, as described in the manuscript.
Population characteristics	For AD patients and healthy controls, people aged 20 or over who live in Japan were eligible as participants in the study. The detail of age and sex information is shown in Supplementary Table 3.
Recruitment	AD patients were recruited in Keio University hospital and healthy controls were recruited in either Keio University or Riken through information posters and documents. Pregnant or breast-feeding women, patients with episodes of lidocaine allergy, prilocaine allergy, or complications of bleeding disorders were excluded from recruitment.
Ethics oversight	The study protocol was approved by the Keio University School of Medicine Ethics Committee and the RIKEN Ethics Committee.

Note that full information on the approval of the study protocol must also be provided in the manuscript.

Field-specific reporting

Please select the one below that is the best fit for your research. If you are not sure, read the appropriate sections before making your selection.

☒ Life sciences ☐ Behavioural & social sciences ☐ Ecological, evolutionary & environmental sciences

For a reference copy of the document with all sections, see nature.com/documents/nr-reporting-summary-flat.pdf

Life sciences study design

All studies must disclose on these points even when the disclosure is negative.

Sample size	No sample size calculations were performed. As a first exploratory study on patient endotypic heterogeneity, sample size were set larger than previous studies on AD based on transcriptome analysis (PMID 33453290, PMID 32615169, PMID 35182548).
Data exclusions	Patients were excluded if they were under systemic therapy with anti-IL-4Rα mAb nor JAK inhibitors. For skin tissue/PBMC RNA-seq, we filtered samples by sequence quality criteria including low total read count and strong batch effect attributable to inadequate sample processing. Skin sample were further filtered by gene expression intensity of pilosebaceous unit-related genes. Detail of our quality control procedures are provided in the methods section of the manuscript.
Replication	No replication of sample was made because replicative sampling of biospecimens from individual subjects is not recommended ethically.
Randomization	No randomization of subjects was performed since this study is an observational study without predefined patient groups.
Blinding	No blinding was performed in data collection nor analysis since this study is an observational study without predefined patient groups.

Reporting for specific materials, systems and methods

We require information from authors about some types of materials, experimental systems and methods used in many studies. Here, indicate whether each material, system or method listed is relevant to your study. If you are not sure if a list item applies to your research, read the appropriate section before selecting a response.

Materials & experimental systems

n/a	Involved in the study
☐	☒ Antibodies
☒	☐ Eukaryotic cell lines
☒	☐ Palaeontology and archaeology
☒	☐ Animals and other organisms
☒	☐ Clinical data
☒	☐ Dual use research of concern

Methods

n/a	Involved in the study
☒	☐ ChIP-seq
☒	☐ Flow cytometry
☒	☐ MRI-based neuroimaging

Antibodies

Antibodies used

For immunohistochemistry, the following antibodies were used: CD4 (Clone 13B8.2, Novus Biological, #NBP2-52670), Myeloperoxidase (Polyclonal, Dako, #A0398), Major basic protein (Clone BMK-13, Bio-Rad, #MCA5751), CD206 (Clone 15-2, Novus biologicals, #NB600-1415), CD11c (Clone B-ly6, BD, #550375), CD1a (Clone O10+C1A/711, Novus biologicals, #NBP2-34314), Keratin 16 (Clone LL025, LabVision, #MS-620-P1), Filaggrin (Clone FLG01, GeneTex, #GTX23137), CD31 (JC/70A, clone Novus biologicals, #NB600-562), FceR1a (clone CRA1, Bio Academia, #72-003), CD208 (clone 104.G4, Immunotech, #IM3448), 2D7 (clone 2D7, Abcam, #ab155577), CD8 (clone C8/144B, Dako, #M710301-2), Lactoferrin (clone B97, Thermo fisher, #14-6604-82).

Validation

The following samples were used to validate the antibodies according to the manufacturers: human lymphocyte for CD4, human acute myeloid leukemia tissue for Myeloperoxidase, human bronchi and skin for Major basic protein, human tonsil for CD206, human spleen for CD11c, human skin for CD1a, human squamous cell carcinoma for Keratin 16, human skin for Filaggrin, human tonsil for CD31, U266 cells for FceR1a, NIH-DC.35 cells for CD208, human nasal mucosa for 2D7, human spleen and tonsil for CD8, human invasive ductal carcinoma for Lactoferrin.
